# Aqueous solutions of super reduced polyoxotungstates as electron storage systems†

**DOI:** 10.1039/d3ee00569k

**Published:** 2023-04-21

**Authors:** Tingting Zhao, Nicola L. Bell, Greig Chisholm, Balamurugan Kandasamy, De-Liang Long, Leroy Cronin

**Affiliations:** a School of Chemistry, University of Glasgow University Avenue Glasgow G12 8QQ UK lee.cronin@glasgow.ac.uk

## Abstract

Due to the increasing energy density demands of battery technology, it is vital to develop electrolytes with high electron storage capacity. Polyoxometalate (POM) clusters can act as electron sponges, storing and releasing multiple electrons and have potential as electron storage electrolytes for flow batteries. Despite this rational design of clusters for high storage ability can not yet be achieved as little is known about the features influencing storage ability. Here we report that the large POM clusters, {P_5_W_30_} and {P_8_W_48_}, can store up to 23 e^−^ and 28 e^−^ per cluster in acidic aqueous solution, respectively. Our investigations reveal key structural and speciation factors influencing the improved behaviour of these POMs over those previously reported (P_2_W_18_). We show, using NMR and MS, that for these polyoxotungstates hydrolysis equilibria for the different tungstate salts is key to explaining unexpected storage trends while the performance limit for {P_5_W_30_} and {P_8_W_48_}, can be attributed to unavoidable hydrogen generation, evidenced by GC. NMR spectroscopy, in combination with the MS analysis, provided experimental evidence for a cation/proton exchange process during the reduction/reoxidation process of {P_5_W_30_} which likely occurs due to this hydrogen generation. Our study offers a deeper understanding of the factors affecting the electron storage ability of POMs and provides insights allowing for further development of these materials for energy storage.

## Abstract 2

The cation/proton exchange illustration during the reduction/reoxidation process of {P_5_W_30_}.

## Graphical abstract

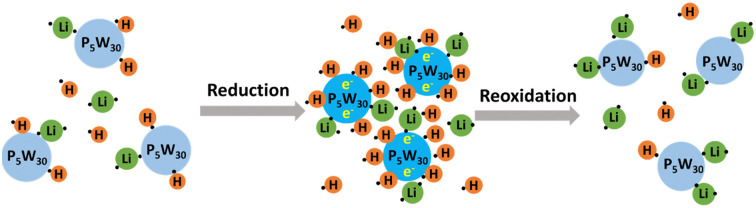

**Broader context**

The storage of electrons by liquid electrolytes is the critical component of a flow battery. The material needs to be stable, safe, and to store as much charge as possible without loss. The benefits of flow batteries include the ability to recharge them by removing the liquid electrolyte and the potential for coupling them into chemical processes. Traditional flow battery electrolytes have been either simple salts or organic molecules, but these can only store 1–2 electrons per molecule and either suffer from stability issues or are corrosive. Recently it was postulated that high nuclearity polyoxometalate clusters could be used to store a large number of electrons per molecule in aqueous solution. In this study we show that large POM clusters, {P_5_W_30_} and {P_8_W_48_}, can store more than 20 electrons per cluster and we explore the mechanism of electron uptake as well as characterize the solutions as potential electrolytes suitable for use in flow batteries.

## Introduction

The transition to a green economy necessitates the development of novel energy storage solutions and there is currently a great deal of interest in redox flow batteries (RFBs) for this application.^1^ Vanadium metal oxides have dominated the field however their commercial development has been limited by their low energy density.^2^ Two key features are salient when optimising RFB performance: (1) potential difference between the materials oxidised and reduced and (2) storage capacity.^3,4^ Significant work has been undertaken to address the former using different redox pairs.^5^ Maximising capacity, or the number of electrons able to be stored and released by a material is still an important area of development.^6^ Polyoxometalates (POMs), are a class of early transition metal oxide clusters with MO_*x*_ (*x* = 5, 6) as basic construction units, which can both store and release multiple electrons due to the reduction and oxidation of high-valence transition metals, like W, Mo and V.^7–10^ Polyoxometalates are highly soluble yielding tungsten concentrations of up to 8 M for PW_12_. Hence, investigating how POMs store and release electrons, and how this relates to their structure, will allow for the further exploitation of their unique properties.

The exceptional electron storage ability of POMs was first hinted at when Launay reported the reduction of [H_2_W_12_O_40_]^6−^, suggesting the reduction of the cluster by six electrons was also accompanied by the transfer of six protons.^11,12^ Bond *et al.* also reported the reduction of the Wells–Dawson [S_2_Mo_18_O_62_]^4−^ anion in a mixture of acetonitrile and water solution, indicating eight reversible one-electron reductions of this anion.^13^ A leap to 24 electron reduction was achieved by Yoshikawa and Awaga, who investigated a Keggin-type POM, [PMo_12_O_40_]^3−^ as a cathode active material.^14^ Through X-ray absorption near-edge structure technique analysis they found all 12 Mo^6+^ centres in this Keggin structure were reduced to Mo^4+^ in the discharging process, which means this Keggin can store 24 e^−^ per cluster. But this performance was achieved in the solid state and not aqueous solution. Since many energy storage devices use aqueous solutions as electrolytes, understanding the electron storage ability of POMs in aqueous solution is important.^1,15^ Normally in aqueous solution, the electron storage ability is limited by side reactions, like hydrogen evolution, as the reduction of these polyoxoanions occurs at only slightly less cathodic potentials than hydrogen evolution as more electrons are put into system. This means it becomes harder to further reduce the POMs and side reactions become an even larger problem.

In previous work we demonstrated that the POM cluster, Li_6_[P_2_W_18_O_62_], has exceptional proton-coupled electron redox activity and can reversibly accept up to 18 protons and electrons in aqueous solution at high concentration and low pH.^16,17^ It is therefore of interest to examine whether this concentration and pH effect applies to other POMs offering the opportunity to increase the storage capacity by utilizing larger clusters. Also, inspired by this initial finding, it is important to investigate if electron storage potential can scale with cluster nuclearity and what properties of a cluster promote electron storage. Therefore, we selected two other phosphotungstates, [NaP_5_W_30_O_110_]^14−^ (abbreviated {P_5_W_30_}) and [H_7_P_8_W_48_O_184_]^33−^ (abbreviated {P_8_W_48_}). {P_5_W_30_}, known as the Preyssler anion, has approximate *D*_5h_ symmetry and consists of a cyclic assembly of five {PW_6_O_22_} units. {P_5_W_30_} is stable within the pH window from 0 to 11 and is reducible to a blue-coloured species, which make it a promising candidate for multi-electron storage.^18–20^ {P_8_W_48_} is a wheel-shaped polyoxotungstate which is stable in pH range 1 to 8; it is also a potential candidate for multi-electron storage.^21^ Previous studies show that the electrochemistry of POMs is dependent on the molecular charge, charge density,^22^ cation type, and cation/anion size.^23,24^ Therefore, as {P_5_W_30_} and {P_8_W_48_} have bigger size and higher charge density than {P_2_W_18_}, the effect of these factors on the electron storage ability suggest that they should be great candidates to test our hypothesis to assess how many electrons can be stored in a single cluster molecule.

To explore the mechanism of reduction to form highly-reduced POMs, previous work suggests it is important to consider the aggregation of polyoxoanions in solution. This can be done by small-angle X-ray scattering (SAXS) *e.g.*, for [PW_12_O_40_]^3−^ and [P_2_W_18_O_62_]^6−^.^14,17^ Furthermore, we reasoned that NMR should also be a powerful technique to study the structure in solution as a function of different redox state.^25–27^ For example, ^7^Li NMR has been used to analyse the mechanism of Li-ion battery charging and discharging processes.^25,26^ More recently, NMR was used to monitor POMs cation–anion interaction in solution. Nyman and co-workers investigated ion-pairing between Cs^+^ cations and Nb/Ta POM anions using inversion-recovery ^133^Cs-NMR spectroscopy demonstrating that strong covalent orbital overlap occurs between the alkali metal and the metal oxide ligands.^28,29^ Bloor and Kidd studied the ^39^K NMR chemical shifts in aqueous solutions with different POM anions and concluded that orbital overlap with metal oxides can decrease paramagnetic shielding of the alkali metal ion relative to solvated ions.^30^ However, there is no NMR study to date of the interactions of Li^+^ counter cations with polyoxoanions, nor investigations of their behaviour upon reduction of the metal centre.

Here, we show that both polyoxoanions {P_5_W_30_} and {P_8_W_48_} demonstrate notable proton-coupled electron redox processes, which allow the two clusters to reversibly accept up to 23 and 28 e^−^ per cluster respectively in acidic aqueous solution. The electron storage ability is concentration based and dependent upon the type of counter cations. The excellent electrochemical performance is attributed to both aggregation of reduced polyoxoanions and their protonation in solution, which was studied by ^7^Li and ^1^H NMR. We also found there is performance limit regarding the total W atoms in the structure, for both {P_5_W_30_} and {P_8_W_48_}, and the possible reasons were analysed including cluster decomposition, competitive hydrogen generation and the effect of different metal coordination environments within the POMs. The ability for these clusters to readily be so highly reduced has importance for the development of new energy storage materials as well as new strategies to develop multi-electron storage systems.

## Results and discussion

### Synthesis and cyclic voltammetry

**Fig. 1:**
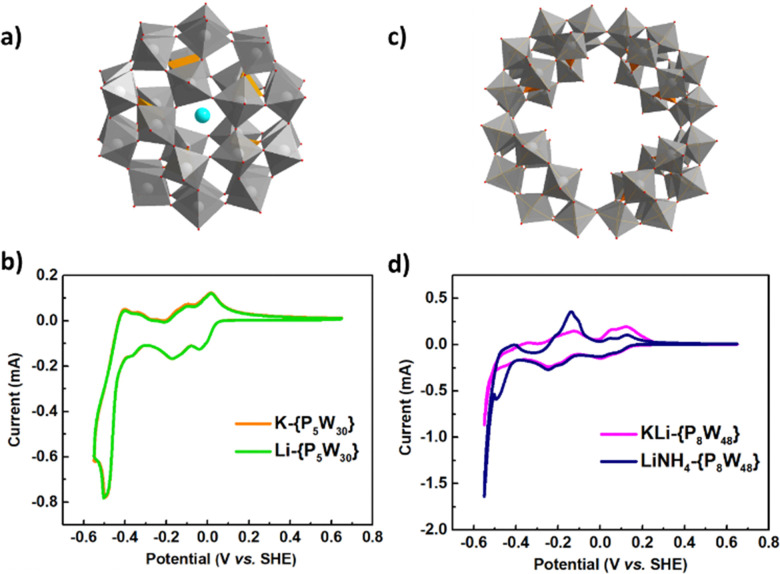
(a) Structure of Li-{P_5_W_30_} by single crystal XRD (grey polyhedra: W; red ball: O; orange polyhedra: P; cyan ball: Na)^18^ (b) cyclic voltammograms of K-{P_5_W_30_} (10 mM) and Li-{P_5_W_30_} (10 mM) in 1 M H_2_SO_4_,^18^ (c) structure of KLi-{P_8_W_48_}^21^ by single crystal XRD (grey polyhedra: W, red ball: O; orange polyhedra: P), (d) cyclic voltammograms of KLi-{P_8_W_48_} (10 mM) and LiNH_4_-{P_8_W_48_} (10 mM) in 1 M H_2_SO_4_.^32^ Scan rate: 10 mV s^−1^. (See ESI† Section 4.1 for further details)

K-{P_5_W_30_} (K_14_[NaP_5_W_30_O_110_]·22H_2_O) was synthesized according to a modified literature procedure.^19,31^ Li-{P_5_W_30_} (Li_14_[NaP_5_W_30_O_110_]·38H_2_O) was synthesized by ion exchange of K-{P_5_W_30_} in excess LiNO_3_ and characterized by single crystal XRD structure determination. Refinement of the diffraction data for Li-{P_5_W_30_} revealed the structure of polyoxoanion of [NaP_5_W_30_O_110_]^14−^, with comparable unit cell parameters to those of K-{P_5_W_30_} (Fig. 1a and Table S4, ESI†).^18^ The redox chemistry of {P_5_W_30_} in sulfuric acid solution was studied by cyclic voltammetry (CV) experiments (Fig. 1b). At low concentration (2 mM), K-{P_5_W_30_} shows four reversible waves within the −0.6 V to 0.6 V range (*versus* standard hydrogen electrode, SHE). Upon moving to higher concentrations of the POM (10 mM), we found there is significant enhancement in the current intensity of the redox waves for K-{P_5_W_30_} when compared with the control study at a concentration of 2 mM (Fig. S15, ESI†) in line with our previous studies.^16^ The CV curves for K-{P_5_W_30_} and Li-{P_5_W_30_} show similar reduction and oxidation waves at 10 mM concentration suggesting that cations may have minimal interaction with the POM in acidic solution.

Similarly, mixed cation salts K_28_Li_5_H_7_[P_8_W_48_O_184_]·92H_2_O (KLi-{P_8_W_48_}) and Li_17_(NH_4_)_21_H_2_[P_8_W_48_O_184_]·85H_2_O (LiNH_4_-{P_8_W_48_}) were synthesized according to modified literature procedures and the structure of KLi-{P_8_W_48_} is shown in Fig. 1c.^21^ CV curves of KLi-{P_8_W_48_} and LiNH_4_-{P_8_W_48_} (Fig. 1d) show different wave shapes, indicating potential different redox mechanisms, with the LiNH_4_-{P_8_W_48_} salt displaying similar reduction behaviour down to −0.4 V but slightly different oxidation behaviour *cf.* the KLi salt, suggesting different cation incorporation, modulating behaviour, upon reduction including hydrogen evolution which will be further detailed below.^32^ However, many of the differences, including the large oxidation peak at ca −0.15 V likely arise from the presence of [NH_4_^+^] ions.

### Flow-cell testing

Previous studies showed the storage of multiple electrons in Li-{P_2_W_18_} was proton-coupled, and as the concentration of Li-{P_2_W_18_} increased, an increasing number of electrons could be stored. To explore if this trend is followed here we explored the cluster redox performance in a three-electrode electrochemical flow cell with an Hg/HgSO_4_ as a reference electrode.^16^ This device can quantify the number of electrons that the polyoxometalate solutions could store. The oxygen evolution reaction (see Fig. S14, ESI†) was used to be the counterpart reaction of the POM reduction reaction. Water is oxidized and simultaneously produces O_2_, protons and electrons (2H_2_O → O_2_ + 4H^+^ + 4e^−^). The protons generated can cross over Nafion membrane and combine with electrons drawn from the external circuit to protonate and reduce an aqueous solution of [NaP_5_W_30_O_110_]^14−^ on the right-hand side of this cell, forming reduced polyoxoanion [NaP_5_W_30_O_110_]^(14+*n*)−^ (*n* = equivalents of electrons charged). Once the desired number of electrons per cluster had been stored, the reduced POM solution was then re-oxidized electrochemically in a neighbour cell on the right-hand side sharing the same POM solution. The electrons stored reversibly in the POM solution could be deduced by comparing the total charge (electrons) input to reduce the POM with the charge (electrons) released out when it was re-oxidized.

### {P_5_W_30_} electron storage capacity studies

**Fig. 2:**
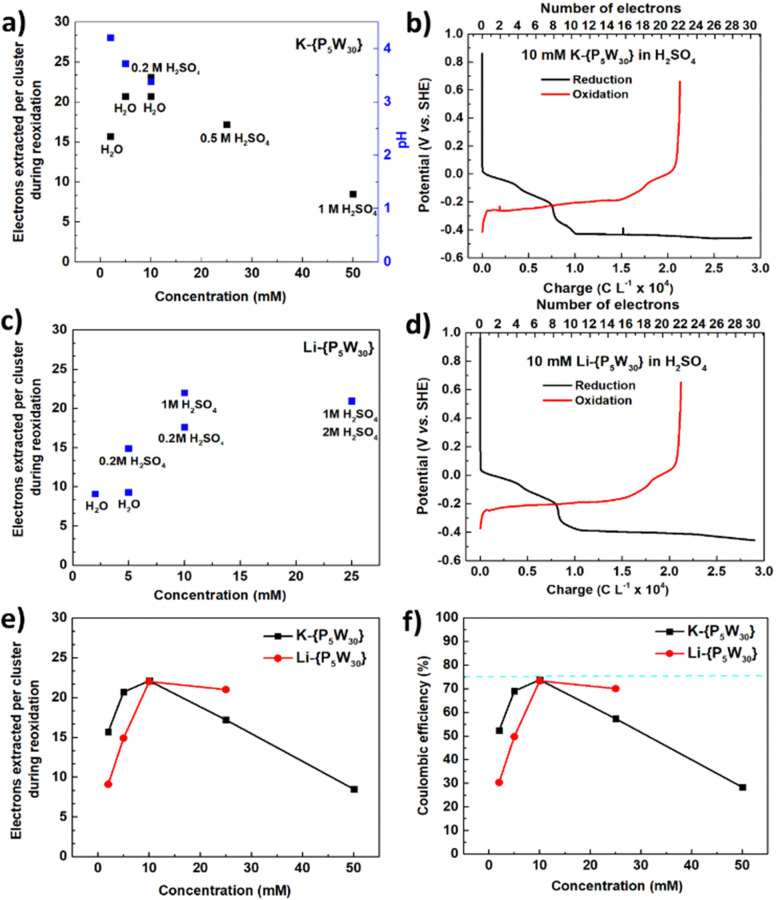
Reversible multi-electron redox chemistry of [NaP_5_W_30_O_110_]^14−^. (a) Relationship between K-{P_5_W_30_} concentration, solution pH (measured before electro-reduction) and the number of electrons that can be extracted from a reduced solution. Solutions were charged 30 electrons. (b) Representative 23 e^−^ reduction/reoxidation curves of a 10 mM solution of K-{P_5_W_30_}, 0.2 M H_2_SO_4_ was used as supporting electrolyte. (c) Relationship between Li-{P_5_W_30_} concentration, and the number of electrons that can be extracted from a reduced solution. (d) Representative 22 e^−^ reduction/reoxidation curves of a 10 mM solution of Li-{P_5_W_30_}, 1 M H_2_SO_4_ was used as supporting electrolyte. In (a) and (c), the number of electrons used to reduce the K-{P_5_W_30_}and Li-{P_5_W_30_} was 30 per cluster. In (b) and (d), a current density of ±10 mA cm^−2^ was applied. A comparison of K-{P_5_W_30_} and Li-{P_5_W_30_}: (e) electrons extracted *vs.* concentration; (f) coulombic efficiency (the total charge extracted out of the total charge put into the POM solutions) *vs.* concentration.

The electron storage ability of K-{P_5_W_30_} was studied *versus* concentration (Fig. 2a and Fig. S16 and Table S5, ESI†). When increasing the concentration from 2 mM to 5 mM in water (the pH decreased from 4.2 to 3.7 accordingly), the electron storage ability of K-{P_5_W_30_} increased from 15.7 e^−^ to 20.7 e^−^. The trend at lower concentration is similar to that observed for the previously reported Li-{P_2_W_18_},^16^ however the trend breaks down above 5 mM as the 10 mM solution (20.7 e^−^) did not show an increase in performance over the 5 mM one (20.7 e^−^). Considering the effect of pH,^33^ we dissolved the cluster in 0.2 M H_2_SO_4_ which led to an increase in the solutions storage capacity to 22.1 e^−^ (a comparison of 10 mM K-{P_5_W_30_} in H_2_O, 0.2 M H_2_SO_4_ and 0.2 M Li_2_SO_4_ can be seen in Fig. S18, ESI†), however upon increasing the concentration further to 25 mM and 50 mM the performance decreased to 17.2 and 8.5 e^−^ respectively. A representative 23 e^−^ reduction/reoxidation curves of a 10 mM solution of K-{P_5_W_30_} in 0.2 M H_2_SO_4_ with the best performance in this group is shown in Fig. 2b.

Lithium salts often have better aqueous solubility than the corresponding potassium salts and some previous studies showed small cations such as Li^+^ can stabilise the reduction of POMs.^23^ Therefore we synthesized Li-{P_5_W_30_} with the expectation this system might show better performance than K-{P_5_W_30_}. However, after conducting flow cell tests under low concentration of 2 mM, it was shown to only store 9.1 e^−^ per cluster, while the 5 mM solution can only store 9.3 e^−^ per cluster (Fig. 2c and Fig. S19 and Table S6, ESI†). We were surprised that the performance is even lower than the K salt given our previous work with Li-P_2_W_18_, but upon investigating the ^31^P NMR of this salt in D_2_O we found that this cluster degraded readily in solution. In contrast a solution in D_2_SO_4_ showed no degradation (Fig. S20, ESI†). Thus, H_2_SO_4_ was used as supporting electrolyte in experiments and the effect of acid was studied. A 5 mM solution of Li-{P_5_W_30_} in 0.2 M H_2_SO_4_ was able to store 14.9 e^−^, while a 10 mM solution was shown to store 18.0 e^−^. Increasing the acid concentration to 1 M while maintaining Li-{P_5_W_30_} at 10 mM allowed storage of up to 22.0 e^−^. At 25 mM substrate in 1 M acid, we found the performance dropped slightly to 21.0 e^−^ and performance was not increased by further increasing acid concentration to 2 M. Thus, it seems these conditions represent the performance limit of Li-{P_5_W_30_}, and the representative reduction/reoxidation curves are shown in Fig. 2d. In total, the electron storage ability of Li-{P_5_W_30_} is affected by both concentrations of POM and supporting acid, increasing with greater concentration of POM from 2 mM to 10 mM and with decreasing pH. However, increasing the concentration further to 25 mM or lowering pH, does not improve the performance more. A comparison of K-{P_5_W_30_} and Li-{P_5_W_30_} with respect to extracted electrons and coulombic efficiency is shown in Fig. 2e and 2f. Both K-{P_5_W_30_} and Li-{P_5_W_30_} show a similar trend of first increasing and then decreasing with a peak performance under 10 mM. In total, K-{P_5_W_30_} is better than Li-{P_5_W_30_} in terms of electron storage ability and coulombic efficiency under lower concentration however Li-{P_5_W_30_} begins to show an advantage under higher concentration.

### {P_8_W_48_} electron storage capacity studies

**Fig. 3:**
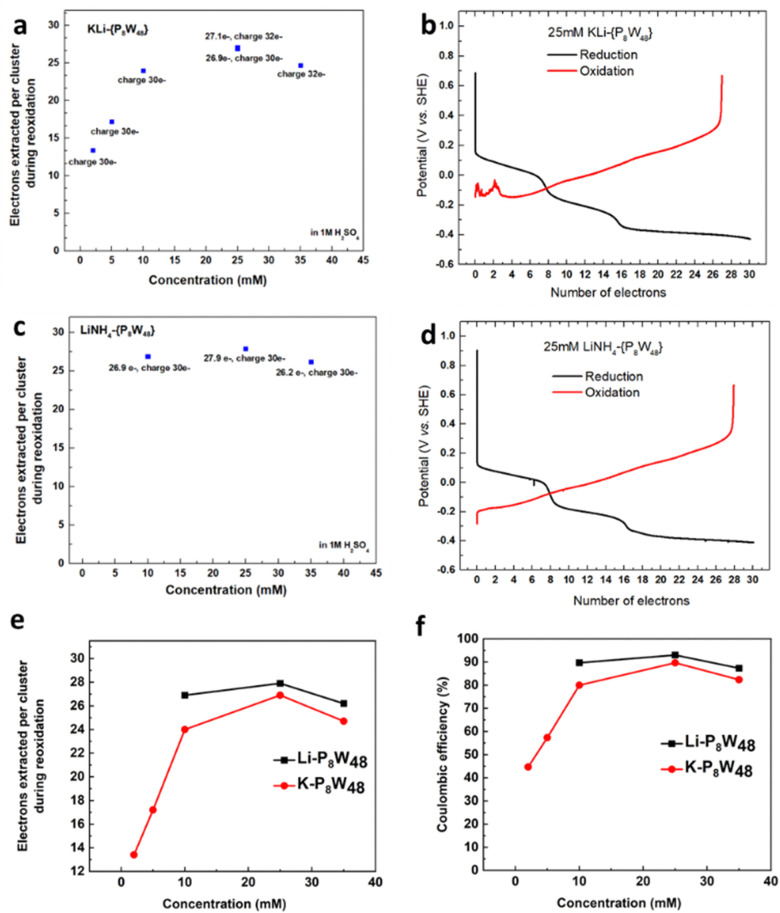
Reversible multi-electron redox chemistry of [H_7_P_8_W_48_O_184_]^33−^. (a) Relationship between KLi-{P_8_W_48_} concentration and the number of electrons that can be extracted from a reduced solution. The number of electrons used to reduce the KLi-{P_8_W_48_} was 30/32 per cluster. 2 mM, 5 mM and 10 mM are under room temperature; 25 mM and 35 mM are under 70 °C. (b) Representative 27 e^−^ reduction/reoxidation curves of a 25 mM solution of KLi-{P_8_W_48_}, 1 M H_2_SO_4_ was used as supporting electrolyte and a current density of ±25 mA cm^−2^ was applied. (c) Relationship between LiNH_4_-{P_8_W_48_} concentration and the number of electrons that can be extracted from a reduced solution. (d) Representative 28 e^−^ reduction/reoxidation curves of a 25 mM solution of LiNH_4_-{P_8_W_48_}. In (b) and (d), a current density of ±25 mA cm^−2^ was applied. A comparison of KLi-{P_8_W_48_} and Li(NH_4_)-{P_8_W_48_}: (e) electrons extracted *vs.* concentration; (f) coulombic efficiency (the total charge extracted out of the total charge put into the POM solutions) *vs.* concentration.

Next the electron storage ability of the larger tungstate wheel, KLi-{P_8_W_48_}, was studied as a function of concentration (Fig. 3a and Fig. S21 and Table S7, ESI†). Here 1 M H_2_SO_4_ was used as the supporting electrolyte for all flow cell tests to overcome the lower solubility of KLi-{P_8_W_48_} in H_2_O. Since {P_2_W_18_} was effectively charged with 18 e^−^ and {P_5_W_30_} was able to be charged with 30 e^−^ we elected to first charging 48 e^−^ per cluster for this salt (*i.e.* assuming each W^VI^ can be reduced to W^V^). At this level of charge the POM shows decomposition with a rapid decrease in electrochemical performance (Fig. S21, ESI,† 25 mM charge 48 e^−^ one). After observing the charge curve carefully, there is an extra step appearing around 28–32 e^−^, so for the following experiment we charged 30 e^−^ per cluster, whereby a normal reoxidation curve was observed. KLi-{P_8_W_48_} showed an increased electron storage ability with an increase in concentration from 2 mM, 5 mM, 10 mM to 25 mM (heating was applied from 25 mM due to the solubility limit under room temperature), when charging 30 e^−^ per cluster, to a maximum of 26.9 e^−^. We then tried to charge more (32 e^−^ per cluster) into this system, the performance did not increase significantly. Increasing the concentration to 35 mM, the performance dropped to 24.7 e^−^. The best performance was achieved at 25 mM with 26.9 e^−^ retrieved after charging by 30 e^−^, shown in Fig. 3b. Similarly, for LiNH_4_-{P_8_W_48_} among 10 mM, 25 mM and 35 mM, the concentration of 25 mM still displays the best performance (27.9 e^−^ out of 30 e^−^, Fig. 3c, d and Table S8 and Fig. S22, ESI†). Overall the LiNH_4_ salt slightly outperformed the KLi salt at all concentrations measured (Fig. 3e).

### Comparison of Li-{P_2_W_18_}, Li-{P_5_W_30_} and LiNH_4_-{P_8_W_48_}

**Fig. 4:**
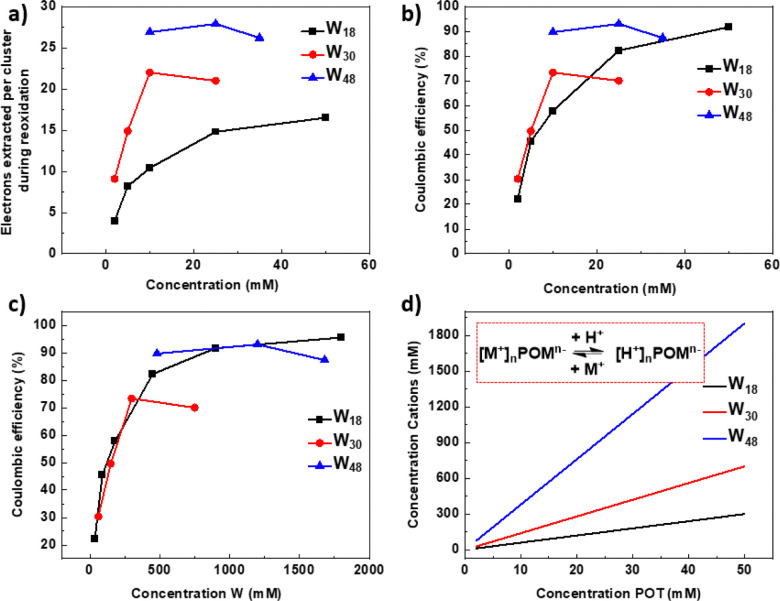
A comparison of electrochemical performance between Li-{P_2_W_18_}, Li-{P_5_W_30_} and LiNH_4_-{P_8_W_48_}. (a) Electrons extracted *vs.* concentration (b) coulombic efficiency *vs.* concentration of polyoxotungstate (POT) (For W_18_, charge 18 e^−^; for W_30_, charge 30 e^−^; for W_48_, charge 30 e^−^, per cluster) (the data for W_18_ are from our prior work^16^). (c) Coulombic efficiency *vs.* concentration of W. (d) Variation of total non-protic counter ions with increasing cluster concentration for the three POMs compared herein. Inset: POM hydrolysis equilibrium between metalation and protonation.

We compared the electrochemical performance of Li-{P_2_W_18_}, Li-{P_5_W_30_} and LiNH_4_-{P_8_W_48_} in terms of extracted number of electrons and coulombic efficiency *versus* concentration for a more comprehensive understanding, shown in Fig. 4a and b. From these two figures, we can see the electron storage ability of POMs is affected by concentration of POMs and their structure among {P_2_W_18_}, {P_5_W_30_} and {P_8_W_48_}. The electron storage ability for these three POMs shows both similarities and differences. In terms of similarities, the electron storage ability of them is all dependent on: (1) the concentration of POMs, (2) the nature of the counter cations and (3) the supporting electrolyte pH or proton concentration.

### Factors influencing performance

The concentration effect for the large clusters P_5_W_30_ and P_8_W_48_ is more complex than for P_2_W_18_, whereby increasing concentration leads to a continuous increase in performance. Instead optimal performance is observed at 10 mM for P_5_W_30_ and 25 mM for P_8_W_48_, although the region between these two concentrations is relatively flat for both (Fig. 4b). For the larger POMs cation concentration increases more rapidly with increasing POM concentration leading to differences in the protonation/metalation equilibrium which occurs through POM hydrolysis in aqueous media (Fig. 4d and Fig. S26, ESI†).^33^ These factors lead to an electron storage performance limit, and even lower performance due to the reduced precipitates which can form during this reduction/reoxidation process, affecting mass transfer in the flow system.

The concentration also affects the impact of different counterions on performance. At low concentration protonation predominates over metalation, particularly for larger, more highly charged cations. Mass Spectrometry data of the two W_30_ salts in both acid and water shows that the potassium salt becomes more protonated than the lithium salt in solution at the same concentration (Fig. S4, S9, S23, S24 and Tables S3, S4, S9, S10, ESI†). This difference in protonation can potentially alter the ability of the POM to stabilise a high charge upon reduction through aggregation. As concentration increases the equilibrium shifts towards metalation, as described above. As such, at high concentration lithium salts show increased performance over Na^+^ and K^+^ salts for all of our POTs (W_18_, W_30_ and W_48_). For {P_8_W_48_}, as both of the samples here have mixed counter cations (K^+^ and Li^+^, Li^+^ and NH_4_^+^), it is hard to analyse the specific effect of a single cation. Mass spectrometry showed poor solubility upon the water dilution required for injection, however we can observe several broad peaks in the envolopes expected for each of the salts. Exemplar spectral windows and simulated patterns for proposed salts can be found in Fig. S41–S43 (ESI†). Importantly, the performance for the Li–(NH_4_)– salt is higher at all concentrations for this cluster.

For coulombic efficiency (Fig. 4b), both {P_5_W_30_} and {P_8_W_48_} showed an advantage over {P_2_W_18_} at low POM concentration (<20 mM), but for {P_2_W_18_}, the coulombic efficiency continues to increase, approaching 100% when concentration reaches 100 mM. It is interesting to note that normalising for W concentration allows us to observe that all three clusters follow a similar efficiency trajectory (Fig. 4c). Regarding the utilization ratio of W atoms (the ratio of W metal centres which are redox-active for storing electrons among all W atoms in the structure), {P_5_W_30_} still showed an advantage at low concentration, but at concentrations higher than 25 mM the advantage of {P_2_W_18_} begins to emerge; {P_8_W_48_} has the lowest utilization rate of W, only part of W in the structure are redox active and participate in the reduction/oxidation process. (Fig. S25, ESI†)

### NMR and mass spectrometry studies

**Fig. 5:**
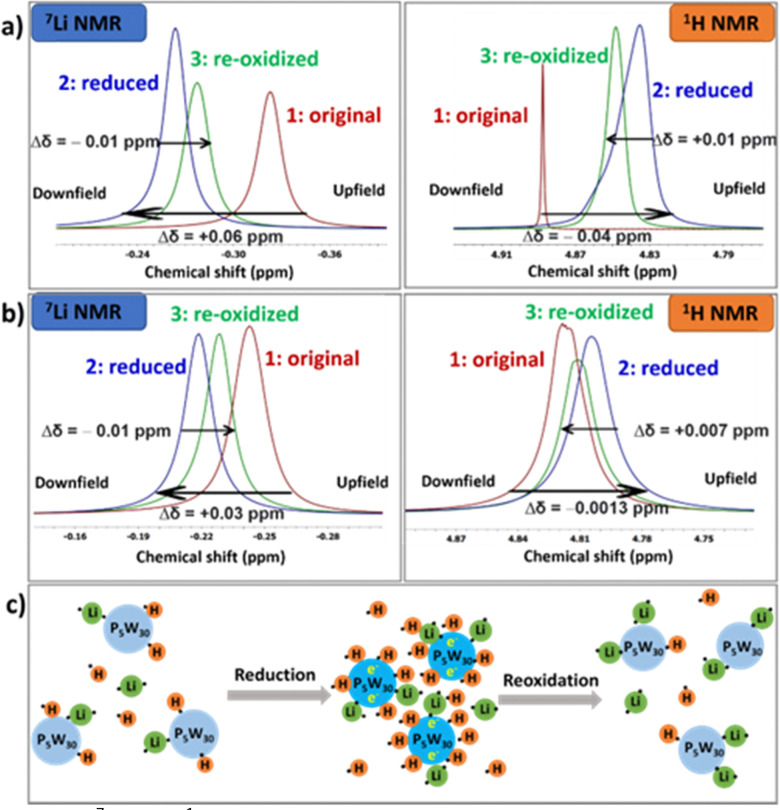
^7^Li and ^1^H NMR study (a) 10 mM of Li-{P_5_W_30_} in 1 M D_2_SO_4_ charge 30 e^−^; (b) 10 mM of Li-{P_5_W_30_} in 1 M H_2_SO_4_ charge 20 e^−^; (c) schematic diagram of protonation/aggregation (reduction and reoxidation process)

NMR and mass spectrometry next allowed us to probe the effect of concentration and reduction/oxidation on speciation, specifically the cation–anion interaction in solution.^29^ Normally ^7^Li NMR shows a sharper signal than ^39^K NMR, so here we use Li-{P_5_W_30_} as an example to study changes in the samples with varying concentration and reduction state through ^7^Li and ^1^H NMR spectroscopy. Firstly, 10 mM of Li-{P_5_W_30_} was studied in 1 M D_2_SO_4_, and both the ^7^Li and ^1^H NMR of original, reduced, and re-oxidized POM solutions were monitored and illustrated in Fig. 5a. Before treatment Li-{P_5_W_30_} resonates at around −0.32 ppm in the ^7^Li spectrum reflecting the mass spectra which show a mixture of lithium and proton cations for the POM in solution (See Fig. S28, ESI†). After reduction, it shifted downfield by +0.06 ppm indicating a decrease in paramagnetic shielding of the lithium ion upon coordination to the POM over solvation^29^ (mass spec suggests all of the fourteen available lithium ions are coordinated). (Tables S12 and S14, ESI†). Li orbitals are overlapping with the POM oxo ligand orbitals which is also consistent with our previous work.^17^ Meanwhile, we monitored ^1^H NMR and an opposite trend compared to ^7^Li NMR was observed. The peak around 4.8 ppm represents the protons from H_2_O (or HOD) which in D_2_SO_4_ solution is in equilibrium with DH_2_O^+^ meaning an intermediate resonance representing the relative position of this equilibrium is observed. After reduction, the acidic protons (deuterons) are bound to the POM oxo ligands shifting this equilibrium and resulting in a more upfield resonance for the H_2_O/HOD protons. The reoxidation process of both ^7^Li and ^1^H showed an opposite trend as the reduction process, but crucially the resonances did not return to the original peak position. (Fig. S26, ESI†) This is due to the fact that production of H_2_ from the system reduces the concentration of H^+^ which shifts the equilibrium towards the formation of H_2_O/HOD (see performance limit section). This altered speciation upon reduction can provide some explanation of the differences in the CV curves shown in Fig. 1d, if we assume the Li- and K- salts have different metalation levels.

Besides ^1^H and ^7^Li NMR, we also monitored ESI-MS and GC for this study and found there is an extra peak on ^31^P NMR at −11.03 ppm after reoxidation (Fig. S27, ESI†), showing the possible partial decomposition of the cluster. From the ESI-MS spectrum, the re-oxidized {P_5_W_30_} has extra peaks at 1091.69 and 1454.92 *m/z*, compared with the original and reduced one. These two peaks can be assigned as species of [H_2_P_2_W_18_O_62_]^4−^ and [H_3_P_2_W_18_O_62_]^3−^, which is consistent with the β-Dawson as shown in NMR spectrum (Fig. S29, ESI†). At the same time, the flow cell test result shows 11.8 e^−^ are extracted after the POM has been charged 30 e^−^ (See Fig. S30, ESI†), which is much less than using H_2_SO_4_ as solvent (22.0 e^−^). Samples of the reduced and reoxidised POM in H_2_SO_4_ showed no extra peaks in the ESI-MS meaning this degradation may occur due to the different rate of reaction of deuterons and protons in electrochemical kinetics. To further confirm the NMR chemical shift trends on ^7^Li and ^1^H, we ran an electrochemical test, charging 20 e^−^ of Li-{P_5_W_30_} in 1 M H_2_SO_4_, and the results are shown in Fig. 5b. Similar trends on both ^7^Li and ^1^H were observed here albeit with a smaller change in chemical shift in both ^7^Li and ^1^H spectra due to the lower charge on the POM. An illustration of this process is shown in Fig. 5c.

We also investigated both the ^7^Li and ^1^H NMR for the original solution with varied concentration from 2 mM, 5 mM, 10 mM, 25 mM to 50 mM for LiCl, Li_6_{P_2_W_18_}, Li_14_{P_5_W_30_} and Li_17_(NH_4_)_21_{P_8_W_48_} (Fig. S31–35, ESI†). While no change in the shift was observed for LiCl over the range (Fig. S31, ESI†) a shift of ^7^Li NMR to downfield was observed when increasing the concentration of all the POMs. This observation supports our assertions above about the effect of increasing alkali metal concentration upon the protonation/metalation equilibrium (effectively decreasing POM hydrolysis). The upfield peak shift in the ^1^H NMR spectrum with an increase in POM concentration is consistent with a similar observation for Li salts in aqueous solution.^34^

### Performance limit analysis

From above electrochemical results, we found that both {P_5_W_30_} and {P_8_W_48_} have an electron extraction limit which is lower than the number of tungsten atoms in the cluster, unlike {P_2_W_18_} which is able to release 17.2 electrons per cluster, with a coulombic efficiency of 96%. Neither {P_5_W_30_} nor {P_8_W_48_} can store 30 e^−^/48 e^−^ per cluster respectively with {P_5_W_30_} storing a maximum of 23 e^−^ per cluster, while {P_8_W_48_} can store 28 e^−^ per cluster. The first possible reason for this performance limit could be POM decomposition during this reduction/reoxidation process. During the electrochemical test, we found that when charging 48 e^−^ per cluster into {P_8_W_48_} the discharge curve rapidly decreased, likely due to degradation of the POM. Secondly, when comparing the structures of the three POMs, we found that the {WO_6_} polyhedra could be either edge-sharing or corner-sharing with their neighbours. In {P_2_W_18_} all of the W atoms are in edge sharing polyhedra whereas in {P_5_W_30_} this number is only 20 and {P_8_W_48_} has 32 edge-sharing polyhedra. Edge sharing polyhedra are known to undergo reduction more easily than their corner sharing counterparts.^35^ Hence a higher barrier to reduction of the corner sharing could limit our ability to charge the cluster. Another possible factor, which may work in concert with the prior issue, could be competitive hydrogen evolution within the electrochemical potential range utilised here.

### POM decomposition

**Fig. 6:**
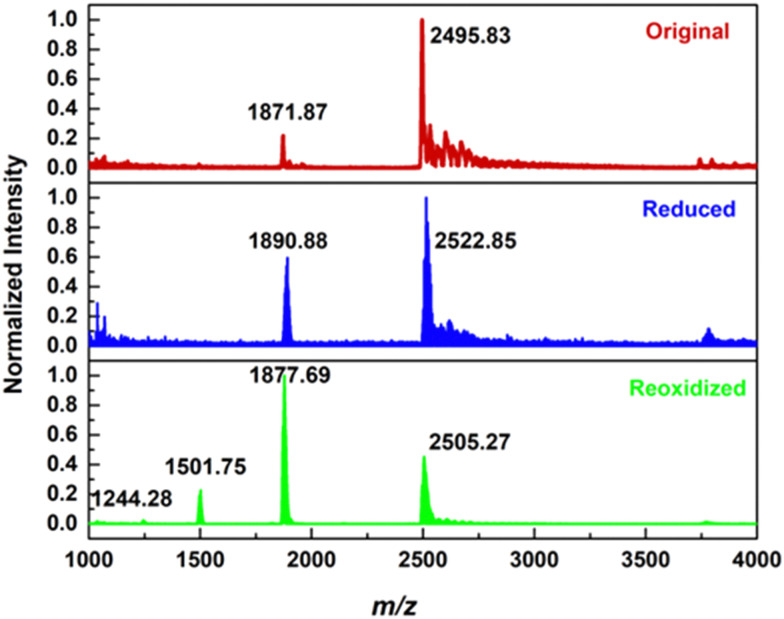
A typical mass spectrum of sample of the polyoxometalate solution taken from a 10 mM solution of Li-{P_5_W_30_} that was (a) original (b) reduced by 30 e^−^ (c) re-oxidized and then injected into the mass spectrometer (after dilution with degassed methanol). The reduced sample was taken out immediately after charging finished and protected with Ar to avoid oxidation by air, before injection it was also diluted with degassed methanol.

We utilised ^31^P NMR and ESI-MS to study the performance limit of Li-{P_5_W_30_} by charging 20, 24 and 30 electrons per cluster respectively into Li-{P_5_W_30_} and monitoring the structure change for the original, reduced and re-oxidized POMs of each. At each of the three charging levels only [NaP_5_W_30_O_110_]^14−^ is observed, indicating there is no decomposition occurring in the experiment (Fig. 6 and Fig. S24, S29, S30, ESI†).

### Hydrogen evolution *vs.* W edge-sharing effect

To analyse for hydrogen evolution and the edge sharing effect we designed a series of three experiments, observing the coulombic efficiency and detection of H_2_. If the edge sharing effect is the principal reason for the performance limit, then when we charge to 20 e^−^ we expect to see 100% coulombic efficiency while if we charge to more than 20 e^−^ we expect to observe hydrogen formation. However, if, no matter how many electrons are added, the coulombic efficiency is always the same (or similar), then hydrogen is formed under all circumstances and the performance limit is caused by unavoidable hydrogen generation.

The electrochemical result and gas analysis for this series of experiment are shown in Table 1. When charged to 30 e^−^, 22 e^−^ was extracted and with H_2_ detected; when charged to 24 e^−^, less electrons (16.4 e^−^) were extracted with H_2_ detected (Fig. S36, ESI†); when charged to 20 e^−^, a significantly lower number of electrons (14.8 e^−^) were released out (Fig. S37, ESI†), with H_2_ still detected. This demonstrates that the performance limit of Li-{P_5_W_30_} is due to unavoidable hydrogen generation rather than the edge-sharing effect. At the same time, we also monitored the structure change (through ESI-MS and ^31^P NMR) for reduced and oxidized ones for charging 20 and 24 e^−^. Consistent results were obtained; no decomposition was observed. (See Fig. S38–S40 and Table S16–S19, ESI†). The reason for this unavoidable hydrogen generation is possibly related to the degree of protonation, the structure of the POM/coordination environment of the W, the charge density of the reduced species and the pH of the battery solution.^16^

**Table 1:** A comparison of battery performance and hydrogen detection of Li-{P_5_W_30_} charge to different electrons (30, 24 and 20 e^−^ per molecule)

	Electrons input	Electrons output	Coulombic efficiency (%)	H_2_ detected
Li-{P_5_W_30_} 10 mM in 1 M H_2_SO_4_	30.0	22.0	73	Yes
	24.0	16.4	68	Yes
	20.0	14.8	74	Yes

## Conclusions

This work shows that polyoxotungstate clusters like M_14_[NaP_5_W_30_O_110_] (M = K^+^, Li^+^) and M_33_[H_7_P_8_W_48_O_184_] (M = K^+^, Li^+^ and NH_4_^+^) can achieve very high proton-electron storage capacities in aqueous solution. {P_5_W_30_} can store up to 23 e^−^ and {P_8_W_48_} can store up to 28 e^−^ per cluster, which is the best electron storage ability reported to date for a single molecule.

However, unlike previously reported {P_2_W_18_} increasing concentration improves storage ability only up to around 10 mM (P_5_W_30_)–25 mM (P_8_W_48_) whereupon the rapidly increasing concentration of cations may impede the ability of the clusters to aggregate through proton bridges. Using acidic electrolyte media can mitigate this effect to a degree and stabilise the POM. At low concentration heavy alkali metal salts (*e.g.* K) show increased storage likely due to cluster hydrolysis however these are surpassed by more electropositive metal salts (*e.g.* Li) as concentration and thus metalation increases. The performance limit in coulombic efficiency for these clusters is caused by unavoidable hydrogen generation. In this regard this work helps understand how polyoxometalates might be exploited for flow batteries and how clusters with greater stability and even higher capacities may be designed.

## Author contributions

L. C. conceived the idea, and L. C and T. Z designed the project. T. Z synthesized {P_5_W_30_} (including all physical characterization), conducted all electrochemistry tests and analysis of {P_5_W_30_} and {P_8_W_48_}, conducted all NMR, MS and GC study of {P_5_W_30_} for mechanisms study and relevant analysis, analysed all data together, and wrote the paper; N. L. B contributed on NMR and MS discussion and analysis and helped write the paper; G. C helped with building up the flow cell and contributed on GC measurement; B. K synthesized {P_8_W_48_} and finished physical characterization of {P_8_W_48_}; D-L. L contributed on single crystal XRD characterization and analysis.

## Conflicts of interest

There are no conflicts to declare.

## Supplementary Material

EE-016-D3EE00569K-s001

EE-016-D3EE00569K-s002
