# Supplementary material for: Aqueous solutions of super reduced polyoxotungstates as electron storage systems
Source: Energy Environ Sci. 2023 Apr 21;16(6):2603–10. doi: 10.1039/d3ee00569k (PMC10265398; doi:10.1039/d3ee00569k)
Supplement: EE-016-D3EE00569K-s001 [file EE-016-D3EE00569K-s001.pdf]

Electronic Supplementary Material (ESI) for Energy & Environmental Science.
This journal is © The Royal Society of Chemistry 2023

Aqueous Solutions of Super Reduced Polyoxotungstates as Electron Storage Systems

Tingting Zhao, Nicola L. Bell, Greig Chisholm, Balamurugan Kandasamy, De-Liang Long and Leroy Cronin*

School of Chemistry, University of Glasgow, University Avenue, Glasgow G12 8QQ, UK.

Table of Contents

1. Materials and instrumentations 2
2. Synthesis and characterization 4
3. Crystallographic analysis of Li-{P_5W_{30}} 12
4. Electrochemical tests 15
  4.1 Cyclic voltammetry 15
  4.2 Flow-cell system for reduction/oxidation of {P_5W_{30}} and {P_8W_{48}} 15
  4.3. Electrochemical performance of K-{P_5W_{30}}, Li-{P_5W_{30}}, KLi-{P_8W_{48}} and LiNH$_4$-{P_8W_{48}} 17
    4.3.1 K-{P_5W_{30}} 17
    4.3.2 Li-{P_5W_{30}} 20
    4.3.3 KLi-{P_8W_{48}} 22
    4.3.4 LiNH$_4$-{P_8W_{48}} 24
5. NMR and ESI-MS analysis of reduction/reoxidation 29
6. Concentration effect of Li-{P_2W_{18}} and Li-{P_5W_{30}} and LiNH$_4$-{P_8W_{48}} 33
7. Performance limit analysis 36
8. References 43

1. Materials and instrumentations

Materials: Sodium tungstate hydrate, phosphoric acid (85 wt. % in H_2O), tris(hydroxymethyl) aminomethane, sodium chloride, potassium chloride, lithium nitrate, lithium acetate, potassium carbonate, methanol, acetic acid, acetone, hydrogen peroxide, sulfuric acid, deuteroxide, sulfuric acid-d2 solution (96-98 wt. % in D_2O, 99.5 atom of D) were purchased from Sigma Aldrich chemicals and Fluka chemicals, respectively. All reagents were purchased and used as received without further treatment.

Elemental Analyses: Element analyses for K, Li, P and were performed on a Leeman inductivity-coupled plasma (ICP) spectrometer and the content of carbon, nitrogen and hydrogen were determined by the microanalysis services using an EA 1110 CHNS, CE-440 Elemental Analyzer.

Single Crystal X-Ray Diffraction: A suitable single crystal was selected and mounted onto a rubber loop using Fomblin oil. Single-crystal datasets and unit cells for compound **Li-{P_5W_{30}}** were collected at 150(2) K on a Rigaku XtaLAB Synergy R HyPix-Arc 150 diffractometer equipped with a graphite monochromator ($\lambda_{Mo\text{-}K\alpha}$ = 0.71073 Å) of a micro-focus sealed X-ray source (50 kV, 24.0 mA). Data collection and reduction were performed using the CrysAlisPro software package and structure solution and refinement were carried out with SHELXT-2018/3 and SHELXL-2018/3 via WinGX.[1] Most of the non-hydrogen atoms were anisotropically refined. Corrections for incident and diffracted beam absorption effects were applied using analytical numeric absorption correction[2] on multifaceted crystal models. CCDC 2173983 contains the supplementary crystallographic data for **Li-{P_5W_{30}}** and can be obtained free of charge via www.ccdc.cam.ac.uk./data_request/cif.

NMR measurements: ^{31}P and ^{7}Li NMR spectra were recorded on a Bruker 400 MHz Lambda/Eclipse spectrometer. ^{1}H NMR spectra were recorded on a Bruker 600 MHz spectrometer and a Bruker 400 MHz Lambda/Eclipse spectrometer respectively. Chemical shifts are referenced at 0 ppm relative to tetramethylsilane for ^{1}H NMR, 9.7 M LiCl solution in D_2O for ^{7}Li NMR and 85% H_3PO_4 for ^{31}P NMR.

ESI-Mass spectrometry: The structure of $[NaP_5W_{30}O_{110}]^{14-}$ were investigated by mass spectroscopy which was collected by using a Bruker MS MaXis Impact instrument (Bruker Daltonics Ltd.). All data were processed using the Bruker Daltonics Data Analysis 4.1 software,

while simulated isotope patterns were investigated using Bruker Isotope software and Molecular Weight Calculator (Lenntech). The calibration solution used was Agilent ESI L low concentration tuning mix solution, product No. G1969-85000, enabling calibration between approximately 500 *m/z* and 4000 *m/z*. Samples were introduced into the MS instrument at a dry gas temperature of 180 °C. The ion polarity for all recorded MS scans was negative, with the voltage of the capillary tip set at 4500 V (3000 V), end plate offset at −500 V, funnel 1RF at 400 Vpp and funnel 2 RF at 400 Vpp, hexapole RF at 400 Vpp (200 Vpp), ion energy −5.0 eV, collision energy at −10.0 eV, collision cell RF at 2100 Vpp (1500 Vpp), transfer time at 120.0 µs, and the pre-pulse storage time at 8.0 µs.

Infrared Spectroscopy: All samples were collected in transmission mode using an ATR fitted JASCO FT-IR-410 spectrometer. Wavenumbers are given in cm^{-1}. Intensities are denoted as w = weak, m = medium, s = strong, br = broad, sh = sharp.

Thermogravimetric Analysis: Analysis for characterization was performed on a TA Instruments Q 500 Thermogravimetric Analyzer under air flow with a heating rate of 10 °C min^{-1} up to 1000 °C.

Gas Chromatography Analysis: Gas chromatography (GC) headspace analysis was performed using an Agilent Technologies 7890A GC system by optimised auto-sampling injection of gas from the headspace of the polyoxometalates holding tank into the GC. The column used was a 30 metre-long 0.320 mm widebore HP-molesieve column (Agilent). the carrier gas was Ar. The GC oven temperature was set to 27 °C and the front inlet was set to 100 °C.

2. Synthesis and characterization

Synthesis of $K_{14}[NaP_5W_{30}O_{110}]\cdot22H_2O$ (1)

$K_{14}[NaP_5W_{30}O_{110}]\cdot22H_2O$ was synthesized from a modified procedure.[3, 4] $Na_2WO_4\cdot2H_2O$ (29.7g, 90 mmol) and NaCl (3.51g, 60 mmol) were dissolved in 125 mL of deionized water in a Teflon-lined autoclave. To this, 21 mL of 85% H_3PO_4 was added, allowing to stir for 1 hr at room temperature. Then, the reaction mixture was heated in an oven heated at 125 °C for 20 h. After cooling, 9 g of KCl (0.12 mmol) was added to the pale-yellow solution and the solution was stirred for 30 min. Then, the pale-yellow solid was separated by centrifugation. Recrystallization was carried by dissolving this solid in 30 mL of deionized water (100 °C, heated in an oil bath). Colourless block crystals were formed in next few days (normally within three days) and collected by filtration. Purer crystals were obtained by recrystallization one more time from 20 mL of deionized water at 100 °C (heated in an oil bath), and the crystals were collected by filtration. The structure of product was confirmed by single crystal XRD unit cell check on multiple crystals and purity was confirmed by ^{31}P NMR (-10.05 ppm in D_2O, Figure S1). The crystal water in the structure was confirmed by TGA (Figure S2). Yield: 6g, based on P/W; ^{31}P NMR (-10.05ppm in D_2O). FT-IR (ATR, 1300-500 cm^{-1}):1157 (sh), 1075 (sh), 1017 (sh), 983 (sh), 903 (sh), 701(br) (Figure S3)

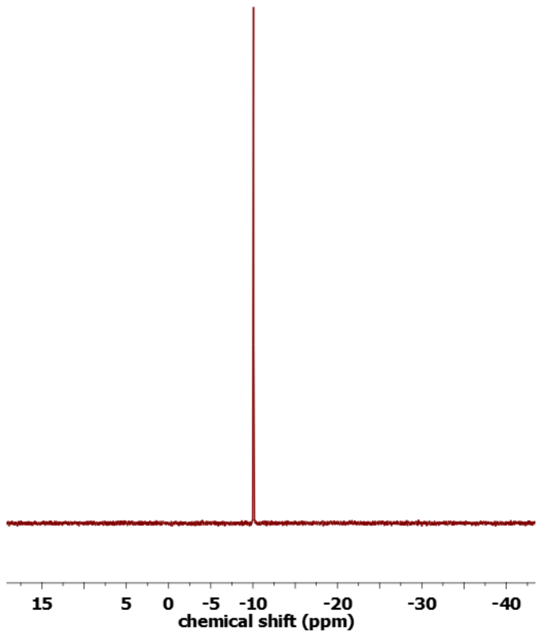

Figure S1: The ^{31}P-NMR spectrum of $K_{14}[NaP_5W_{30}O_{110}]$ in D_2O at 298K.

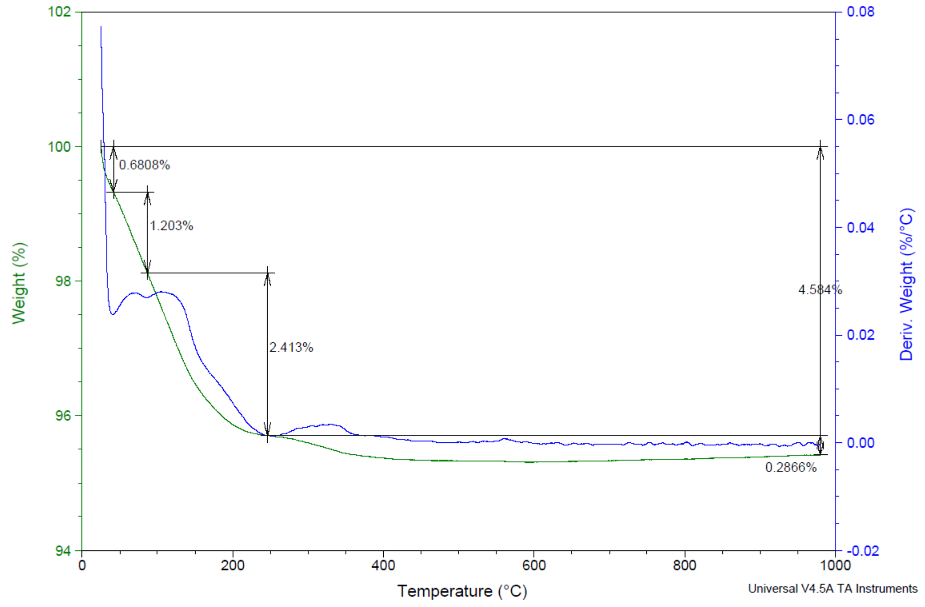

Figure S2: Thermogravimetric analysis of $K_{14}[NaP_5W_{30}O_{110}]\cdot xH_2O$, the loss of mass from room temperature to 1000 °C was used to determine the water content in the crystals.

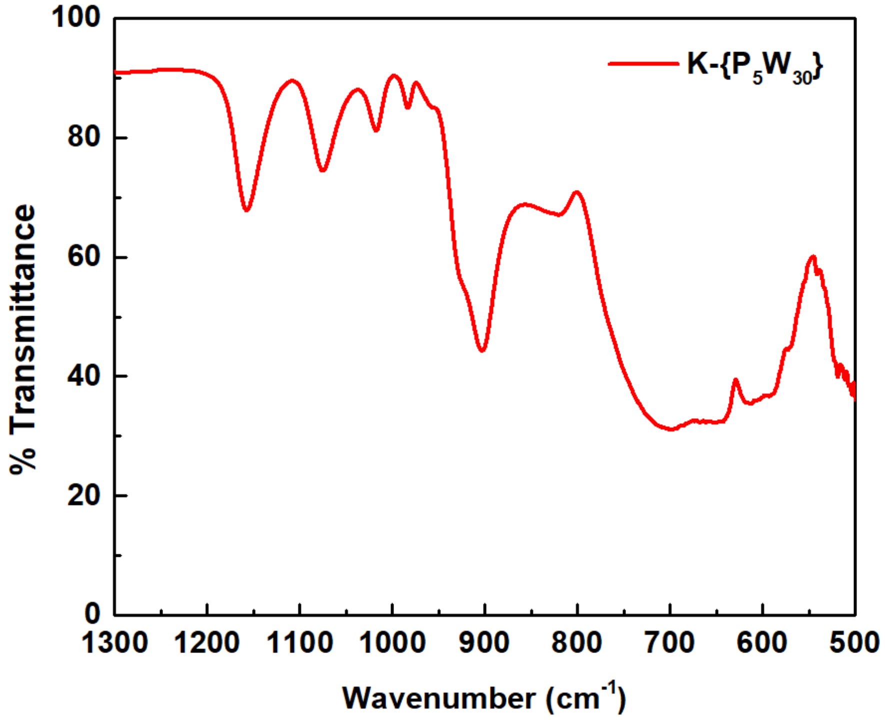

Figure S3: FTIR spectrum of K-{P_5W_{30}} (in solid state)

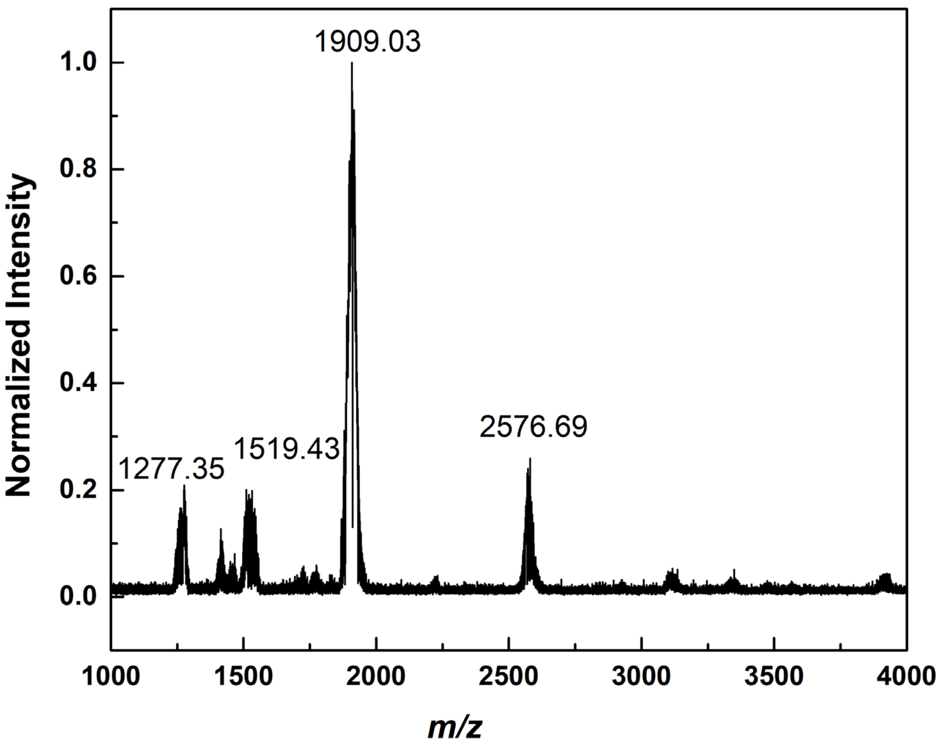

Figure S4 : ESI-MS of $K_{14}[NaP_5W_{30}O_{110}]\cdot 22H_2O$ in water, confirming the structure of $[NaP_5W_{30}O_{110}]^{14-}$.

Table S1: Mass Spectrum peak table for $K_{14}[NaP_5W_{30}O_{110}]$ in water (shown in Figure S4)

z	m/z (Obs)	m/z (Cal)	Assignment
6-	1277.35	1276.23	$[K_4H_2Na_3P_5W_{30}O_{110}]^{6-}$
5-	1519.43	1519.66	$[K_3H_5Na_2P_5W_{30}O_{110}]^{5-}$
4-	1909.03	1909.35	$[K_4H_5Na_2P_5W_{30}O_{110}]^{4-}$
3-	2576.69	2578.86	$[K_6H_3Na_3P_5W_{30}O_{110}]^{3-}$

Synthesis of $Li_{14}[NaP_5W_{30}O_{110}]\cdot 38H_2O$ (2)

$Li_{14}[NaP_5W_{30}O_{110}]\cdot 38H_2O$ was synthesized through lithium ion exchange of $K_{14}[NaP_5W_{30}O_{110}]\cdot 22H_2O$ in $LiNO_3$ saturated solution. A typical synthesis process is 1.89 M solution of $LiNO_3$ in 90 mL of MeOH was added to 13 mL of a 0.018 M solution of $K_{14}[NaP_5W_{30}O_{110}]\cdot 22H_2O$ in water the clear solution was kept stirring for 1h. Then excessive solvent (methanol and water) of the clear solution was removed by rotary evaporation. On next day, colourless crystals formed (morphology see Figure S5). The structure was confirmed by

single crystal XRD and ^{31}P NMR (-10.51 ppm in 1M D_2SO_4, Figure S6), and the composition and purity were checked by ICP and elemental analysis (Table S2). The crystal water was analysed by TGA (Figure S7). Yield: 1.5 g. FTIR (ATR, 1300-500 cm-1): 1162 (sh), 1084 (sh), 1021 (sh), 986 (sh), 902 (sh), 717 (br) (Figure S8). The structure of this sample was further confirmed by ESI-MS spectrum (Figure S9 and Table S3)

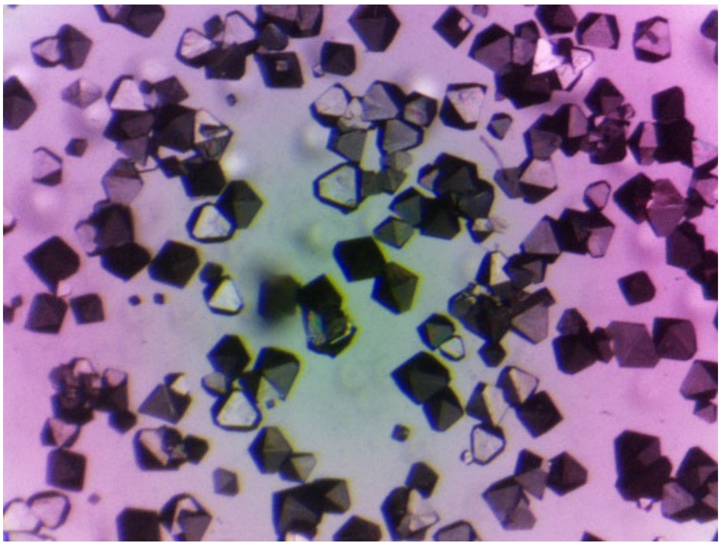

Figure S5: Digital image of $Li_{14}[NaP_5W_{30}O_{110}]\cdot38H_2O$.

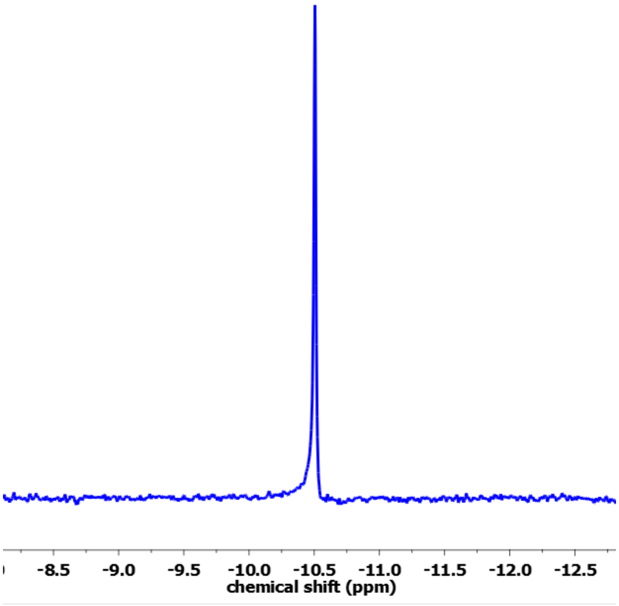

Figure S6: The ^{31}P-NMR spectrum of $Li_{14}[NaP_5W_{30}O_{110}]\cdot38H_2O$ in D_2SO_4 at 298K.

Table S2: ICP analysis of $Li_{14}[NaP_5W_{30}O_{110}]\cdot38H_2O$ showing the atomic ratio of Li and K = 28:1, indicating a complete exchange of Li^+ and K^+

Element	K	Li	Li	Na	Na	P	W
wavelength (nm)	766.491	610.365	670.783	588.995	589.592	213.618	207.912
Result (ppm)	0.24	1.23	1.38	0.35	0.46	1.79	53.64

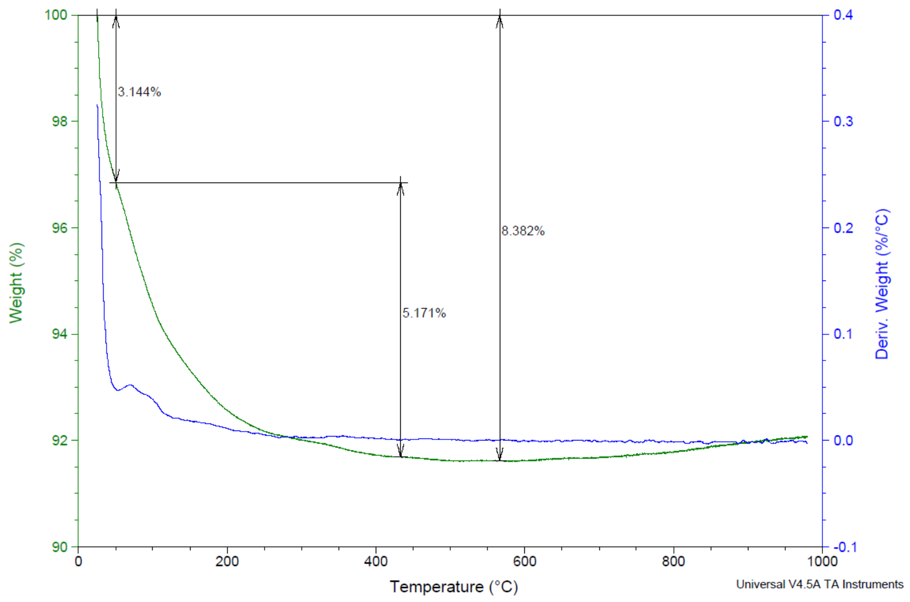

Figure S7: Thermogravimetric analysis of $Li_{14}[NaP_5W_{30}O_{110}]\cdot xH_2O$, the loss of mass (8.38%) from room temperature to 560 °C was used to determine the water content in the crystals.

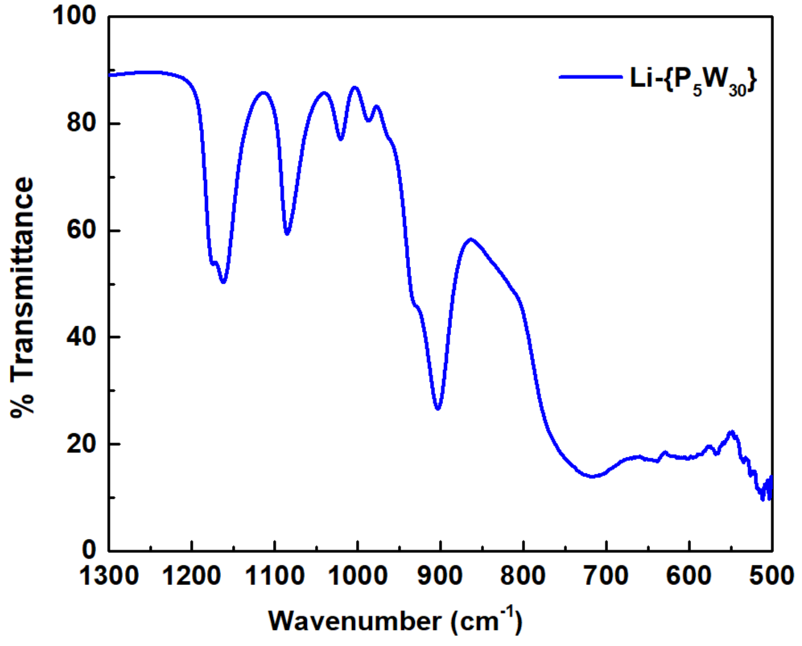

Figure S8: FTIR spectrum of Li-{P_5W_{30}} (in solid state)

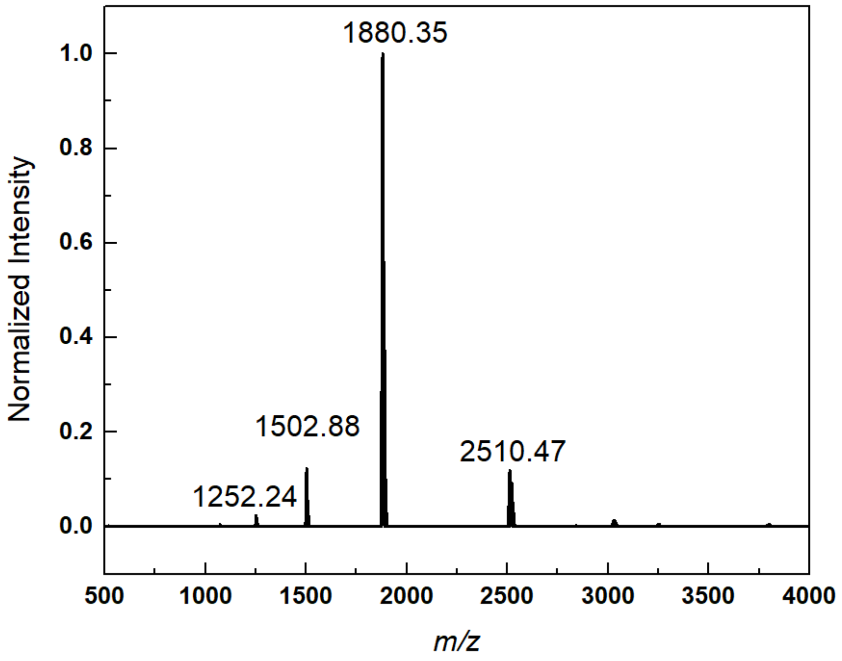

Figure S9: ESI-MS of $Li_{14}[NaP_5W_{30}O_{110}]·38H_2O$ in methanol, confirming the structure of $[NaP_5W_{30}O_{110}]^{14-}$. The ion polarity for all recorded MS scans was in negative mode.

Table S3a: Mass Spectrum peak table for $Li_{14}[NaP_5W_{30}O_{110}]·38H_2O$ in methanol (shown in Figure S9)

z	m/z (Obs)	m/z (Cal)	Assignment
6-	1252.24	1251.49	$[Li_8NaP_5W_{30}O_{110}]^{6-}$
5-	1502.88	1503.10	$[Li_9NaP_5W_{30}O_{110}]^{5-}$
4-	1880.35	1880.60	$[Li_{10}NaP_5W_{30}O_{110}]^{4-}$
3-	2510.47	2509.78	$[Li_{11}NaP_5W_{30}O_{110}]^{3-}$

Table S4b: Mass Spectrum peak table for $Li_{14}[NaP_5W_{30}O_{110}]·38H_2O$ in water

z	m/z (Obs)	m/z (Cal)	Assignment
6-	1251.88	1251.49	$[Li_8NaP_5W_{30}O_{110}]^{6-}$
5-	1505.86	1505.60	$[Li_8HNaP_5W_{30}O_{110}].(H_2O)^{5-}$
4-	1883.82	1883.25	$[Li_9HNaP_5W_{30}O_{110}].(H_2O)^{4-}$
3-	2522.36	2522.01	$[Li_{11}NaP_5W_{30}O_{110}].(H_2O)_2^{3-}$

Synthesis of $K_{28}Li_5H_7[P_8W_{48}O_{184}]\cdot 92H_2O$ (3)

The original synthetic procedure of synthesizing $K_{28}Li_5H_7[P_8W_{48}O_{184}]\cdot 92H_2O$ was reported in the literature[5]. However, we have found that the former method has two important obstacles, namely, yield and prolong time of crystallisation. Therefore, Cronin group has developed new synthetic protocol for the preparation of KLi-$\{P_8W_{48}\}$ derivatives, which was followed for this work. As with the preparation of $K_{12}H_2[\alpha\text{-}P_2W_{12}O_{48}]\cdot 24H_2O$, fresh starting material was used to ensure the optimum yield.

To 200 mL H_2O in a 250 mL beaker, was added 6.0 mL CH_3CO_2H, followed by lithium acetate dihydrate (9.0 g, 88 mmol). After 5 minutes of vigorous stirring to allow the lithium acetate to dissolve, LiCl (4.24 g, 100 mmol) was then added, before again allowing 5 minutes to dissolve. Finally, $K_{12}H_2[\alpha\text{-}P_2W_{12}O_{48}]\cdot 24H_2O$ (5.60 g, 1.42 mmol) was added over a period of 30 minutes, whilst maintaining vigorous stirring. Once the addition was complete, the solution was stirred for ten further minutes, before setting-aside in a temperature-controlled room (18 °C) for crystallisation. The colourless needle shaped crystals were collected after 7 days and used as such without further purification. Yield: 750 mg, 51 μmol, 14.3 % based on P/W. The bulk purity of the KLi-$\{P_8W_{48}\}$ was confirmed by ^{31}P NMR (Figure S10). The major peak was appeared at -7.58 ppm. FT-IR (ATR, 4000-500cm^{-1}): 1136 (m, sh), 1082 (m, sh), 1017 (wk, sh), P-O stretching frequencies. (Figure S12) Elemental analysis for $K_{28}Li_5H_7[P_8W_{48}O_{184}]\cdot 92H_2O$. Mw = 14809.91, calc. (%): H 1.30, Found (%): H 1.28.

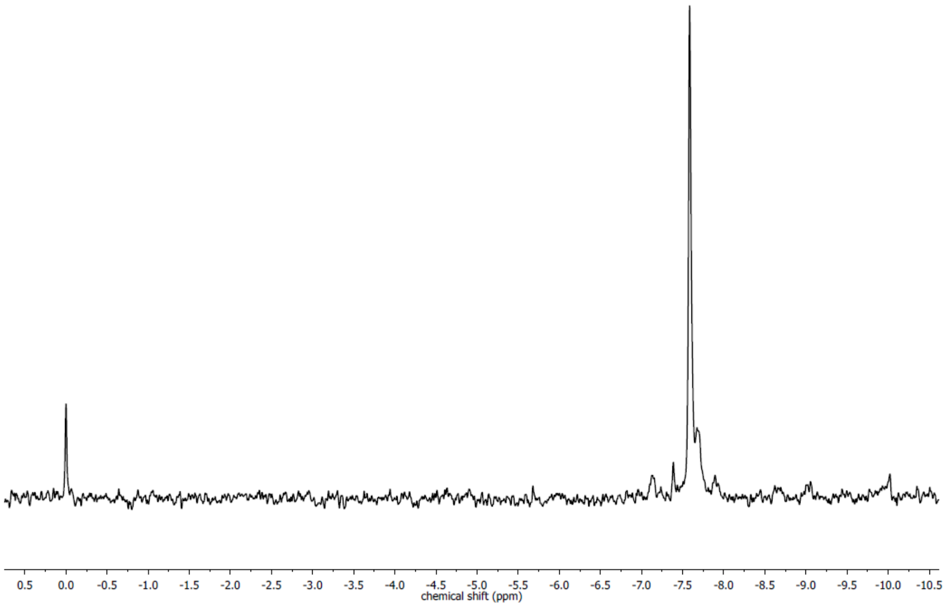

Figure S10: ^{31}P NMR of $K_{28}Li_5H_7[P_8W_{48}O_{184}]\cdot 92H_2O$ in pure $LiCl/D_2O/H_3PO_4$

Synthesis of $Li_{17}(NH_4)_{21}H_2[P_8W_{48}O_{184}]\cdot 85H_2O$ (4)

For the preparation of both $(NH_4)_{12}H_2[\alpha\text{-}P_2W_{12}O_{48}]\cdot 19H_2O$ and $Li_{17}(NH_4)_{21}H_2[P_8W_{48}O_{184}]\cdot 85H_2O$, acid-washed plastic reaction vessels were used to ensure no leaching of potassium from glassware. Again, fresh POM starting material was used to ensure the optimum yield.

To 200 mL H_2O was added firstly LiCl (4.24 g, 0.10 mol), then, once dissolved, lithium acetate dihydrate (10.20 g, 0.10 mol). After the solution had gone clear, $(NH_4)_{12}H_2[\alpha\text{-}P_2W_{12}O_{48}]\cdot 19H_2O$ (5.00 g, 1.39 mmol), which dissolved readily, was added without concern for the rate of addition. Finally, the pH was altered to exactly 5.00 with glacial acetic acid. Large colourless needle-like crystals formed from solution within 3 weeks and were collected by Büchner filtration, dried it in air for overnight. Analysis: Total yield: 0.57 g, 0.0575 mmol, 16.5 % based on W. The bulk purity of the LiNH4-$\{P_8W_{48}\}$ was confirmed by ^{31}P NMR. (Figure S11). The major peak was appeared at -8.29 ppm. FT-IR (ATR, 3800-500 cm^{-1}): 1438 (strong, NH_4^+ cations), 1136 (m, sh), 1082 (m, sh), 1017 (wk, sh), P-O stretching frequencies (Figure S12). Elemental analysis for $Li_{17}(NH_4)_{21}H_2[P_8W_{48}O_{184}]\cdot 85H_2O$, Mw = 14046.11, calc. (%): H 1.84, N 2.09 Found (%): H=1.73, N= 2.21.

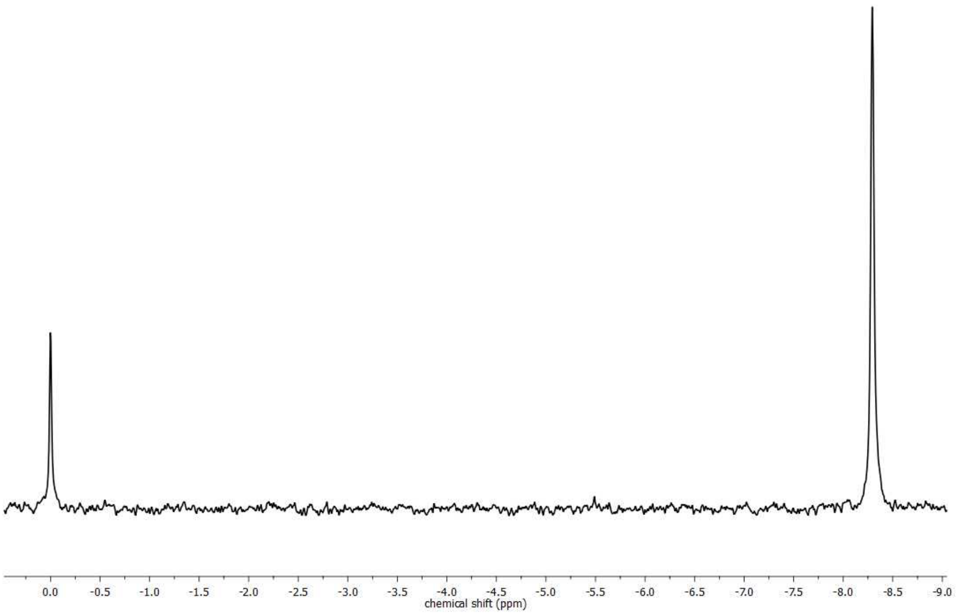

Figure S11: ^{31}P NMR of $Li_{17}(NH_4)_{21}H_2[P_8W_{48}O_{184}]\cdot 85H_2O$ in $LiCl/D_2O/H_3PO_4$.

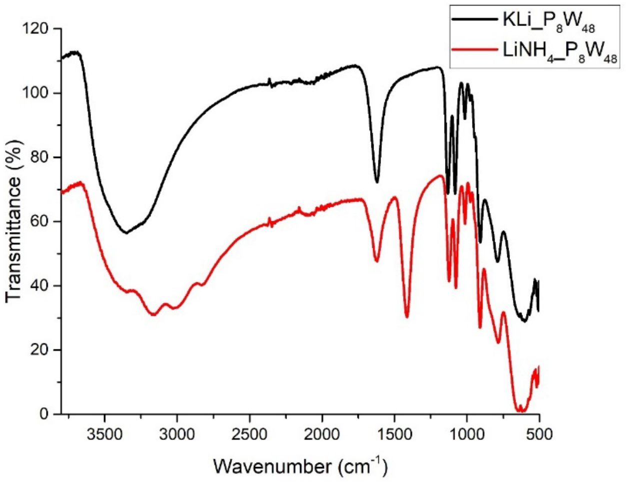

Figure S12: FT-IR of KLi-{P_8W_{48}} and $LiNH_4$-{P_8W_{48}}.

3. Crystallographic analysis of Li-{P_5W_{30}}

Table S5: Crystal data and structure refinement details.

Identification code	$Li_{14}[NaP_5W_{30}O_{110}]\cdot 38H_2O$	
Empirical formula	$H_{76}Li_{14}NaO_{148}P_5W_{30}$	
Formula weight	8235.10	
Temperature	150(2) K	
Wavelength	0.71073 Å	
Crystal system	Monoclinic	
Space group	*C*2/*m*	
Unit cell dimensions	*a* = 27.1590(12) Å	α = 90°.
	b = 21.1739(9) Å	β = 95.848(4)°.
	c = 23.6358(10) Å	γ = 90°.
Volume	13521.3(10) Å^3	
Z	4	
Density (calculated)	4.045 Mg/m^3	
Absorption coefficient	25.591 mm^{-1}	
F(000)	14432	
Crystal size	0.095 x 0.089 x 0.056 mm^3	
θ range for data collection	2.444 to 26.556°.	
Index ranges	-32 <= *h* <= 33, -26 <= *k* <= 26, -29 <= *l* <= 29	
Reflections collected	18143	

Independent reflections	55810 [R(int) = 0.042]
Completeness to theta = 25.242°	99.7 %
Absorption correction	Gaussian
Max. and min. transmission	0.408 and 0.212
Refinement method	Full-matrix least-squares on F^2
Data / restraints / parameters	18143 / 354 / 726
Goodness-of-fit on F^2	1.063
Final R indices [$I > 2\sigma(I)$]	$R1$ = 0.0809, w$R2$ = 0.2189
R indices (all data)	$R1$ = 0.0908, w$R2$ = 0.2268
Extinction coefficient	n/a
Largest diff. peak and hole	8.33 and -2.81 e.Å^{-3}

$[NaP_5W_{30}O_{110}]^{14-}$ cluster displays a round plate shape and has a symmetry of D_{5h} point group (Figure S13). A fivefold axis passes the Na atom and vertical to plate equatorial plane defined by the five P atoms. The lithium salt compound **2** $Li_{14}[NaP_5W_{30}O_{110}]$.38H2O crystallises in monoclinic system *C*2/*m* space group. Half $\{P_5Mo_{30}\}$ cluster was found in the asymmetric unit. The cluster is completed with the other half cluster generated by a crystallographic mirror plane, which is also the horizontal (equatorial) plane symmetry (σ_h) of the cluster D_{5h} point group. The Na atom inside the cluster is disordered over two positions each with half occupancy. The structure is refined as a 2-component twin. Due to being twined, the final structure mode contains some large positive residual densities (max. 8.33 eÅ^{-3}) appearing on positions unresolvable for structural atoms. No attempt was made to allocate Li positions in the refinement of this heavy metal tungsten structure. SQUEEZE was applied to calculate the void space, the electron counts for disordered solvent and cation and to produce new HKL files for further structure refinements. The formula was determined with the cluster found in structure refinement and Li and solvate water content from chemical analysis ICP and TGA.

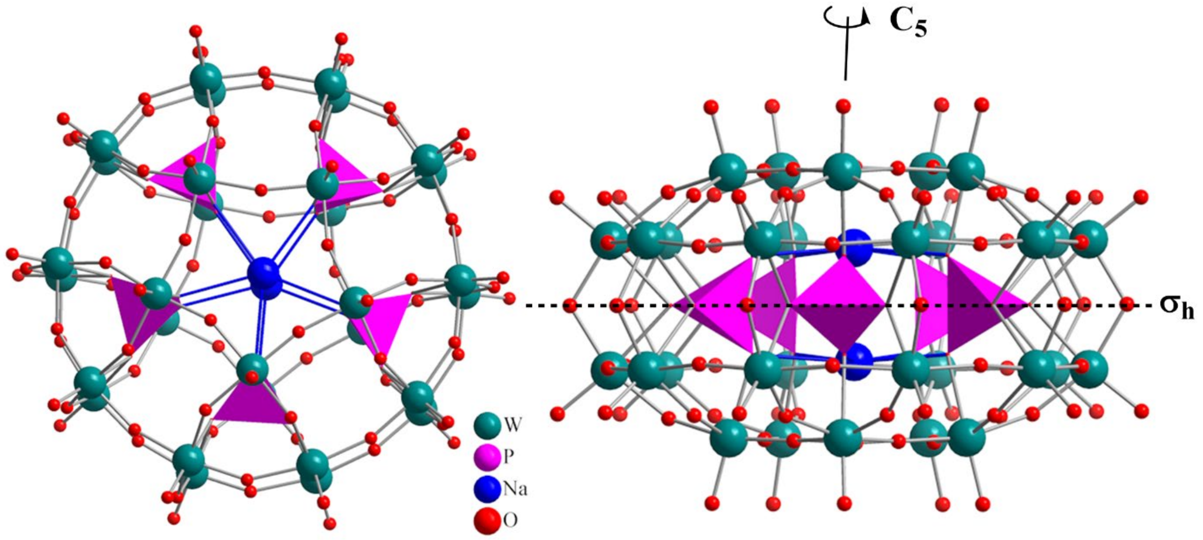

Figure S13: Down and side views of the $[NaP_5W_{30}O_{110}]^{14-}$ cluster. Colour scheme: W cyan, Na blue, O red, PO_4^{3-} pink polyhedron.

4. Electrochemical tests

4.1 Cyclic voltammetry

All the electrochemical data were collected by using a Bio-logic SP-150 potentiostat. A home-made electrochemical cell was used for cyclic voltammetry (CV) measurements. A glassy carbon electrode, carbon felt and $Hg/HgSO_4$ (sat. K_2SO_4) were used as the working electrode, counter electrode, and reference electrode respectively. All the solutions were degassed with Ar for at least half an hour to remove the oxygen before CVs were collected. The scan rate is 10 mV s^{-1}.

4.2 Flow-cell system for reduction/oxidation of $\{P_5W_{30}\}$ and $\{P_8W_{48}\}$

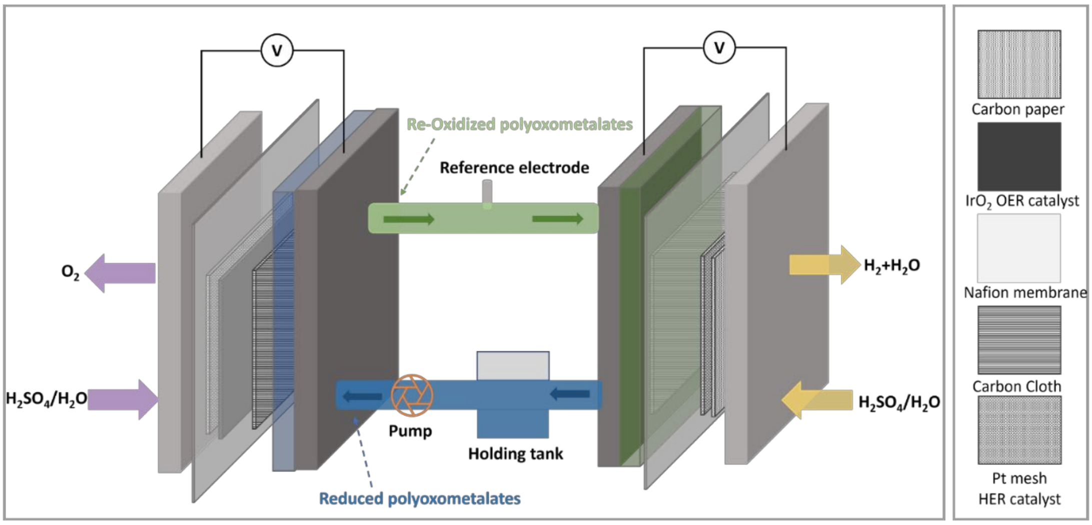

Figure S14: Flow cell device used for the reduction and oxidation of $\{P_5W_{30}\}$ and $\{P_8W_{48}\}$. HER, hydrogen evolution reaction; OER, oxygen evolution reaction.

The two-parts flow cell system comprised a mixture of commercial and custom-made components. It was assembled in Figure S14, in similar way described in our previous paper.[6] POCO graphite plates (thickness = 3mm, channel width = 1 mm, channel depth = 1 mm, landing between channels = 1 mm) were purchased from balticFuelCells GmbH and were cleaned by heating with concentrated HNO_3 (70%) at 80 °C for 1 h to 2 h. Two layers of carbon cloth were pre-treated by soaking in H_2SO_4: HNO_3 (3:1 v/v) for 3h and rinsed with and stored in ultra-pure water for use as diffusion layers. These components were used as the electrodes for reduction and oxidation of solutions of $\{P_5W_{30}\}$ and $\{P_8W_{48}\}$. An extra stainless steel foil was used as the current collector on this side of the cell.

The Nafion membrane was pre-treated according to published procedures[7]. Two layers of Nafion membrane (Nafion 117, supplied by Ion Power) were boiled in 3% H_2O_2 at 80 °C for 1 h, washed with ultra-pure water, then immersed in 1 M H_2SO_4 at 80 °C for 1 h, and finally rinsed with water. The electrodes where the oxygen and hydrogen evolution reactions occur were prepared as follows. One layer of Toray carbon paper (TCP-H-60, supplied by Fuel Cell Etc., pre-treated as above) and an iridium oxide-coated membrane (Nafion 115, one side coated, supplied by Ion Power) was used as diffusion layer and catalysts for the oxygen evolution reaction. Similarly, one layer of Toray carbon paper and a Pt mesh were used for hydrogen evolution reaction. The current collectors for these sides were 12.96 cm^2 titanium blocks with flow channels (thickness = 3 mm, channel width = 1 mm, channel depth = 1 mm, landing between channels = 1 mm) purchased from Glenhead Engineering Ltd., UK.

Metallic aluminium and PTFE plates with machined holes were used as flow cell endplates for both sides of the flow cells. Eight bolts (M6 × 60 mm Full Thread Hexagon Bolts, RS-Components, UK) were used to tight and complete the cell assembly (with a torque of 6 Nm). PTFE tubes were used to transport electrolyte. Pumps were used to circulate the fluids at a flow rate of 100 mL min^{-1} to minimise mass transport overpotentials. All the electrochemical data for flow cells were collected using a Bio-logic SP-150 potentiostat. POM redox and oxidation reactions were performed while keeping POM solution under Ar atmosphere. Also, the POM solutions were degassed with Ar for at least half an hour to remove the oxygen before reduction (oxidation) data were collected. For flow cell tests of $\{P_8W_{48}\}$, solubility only can be achieved by temperature, hence a modification with heating on this device system (POM electrochemical vessel and OER/HER electrolyte tanks) were completed.

Such a device has a very low ohmic polarization resistance of around 50 to 100 mΩ. Galvanostatic electrolysis method with a suitable current density was applied to reduce the polyoxometalate solution. The voltage plateaus in the potential verse charge curves during the reduction (and oxidation) processes correspond to the thermodynamic potentials for the different amount of electrons put into this system.

4.3. Electrochemical performance of K-{P_5W_{30}}, Li-{P_5W_{30}}, KLi-{P_8W_{48}} and LiNH$_4$-{P_8W_{48}}

4.3.1 K-{P_5W_{30}}

The electrochemical performance of K-{P_5W_{30}} was evaluated by cyclic voltammetry under different concentrations (2 mM and 10 mM). (Figure S15)

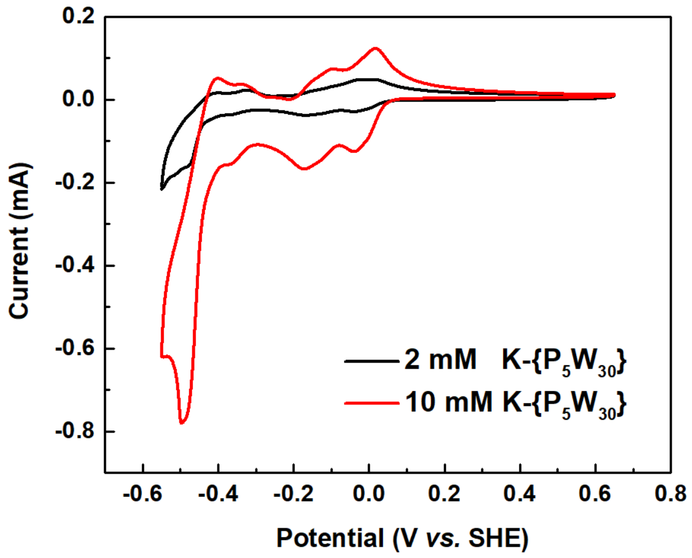

Figure S15: Cyclic voltammograms of K-{P_5W_{30}} (2 mM and 10 mM), in 1 M H_2SO_4, scan rate 10 mV s^{-1}

Table S6: Comparison of the number of electrons that can be stored in K-{P_5W_{30}} at different concentrations in H_2O/H_2SO_4. In all cases, the number of electrons used to initially reduce the K-{P_5W_{30}} was set at 30 per cluster.

Conc. K-{P_5W_{30}} (mM)	2	5	10	10	25	50
Volume (mL)	25	25	25	25	25	25
Supporting electrolyte	/	/	/	0.2 M H_2SO_4	0.5 M H_2SO_4	1 M H_2SO_4
Current density (mA cm^{-2})	2	5	10	10	25	50
Coulombic efficiency (%)	52.3	69.0	69.0	77.0	57.3	28.3
Number of electrons stored per cluster	15.7	20.7	20.7	22.1	17.2	8.5

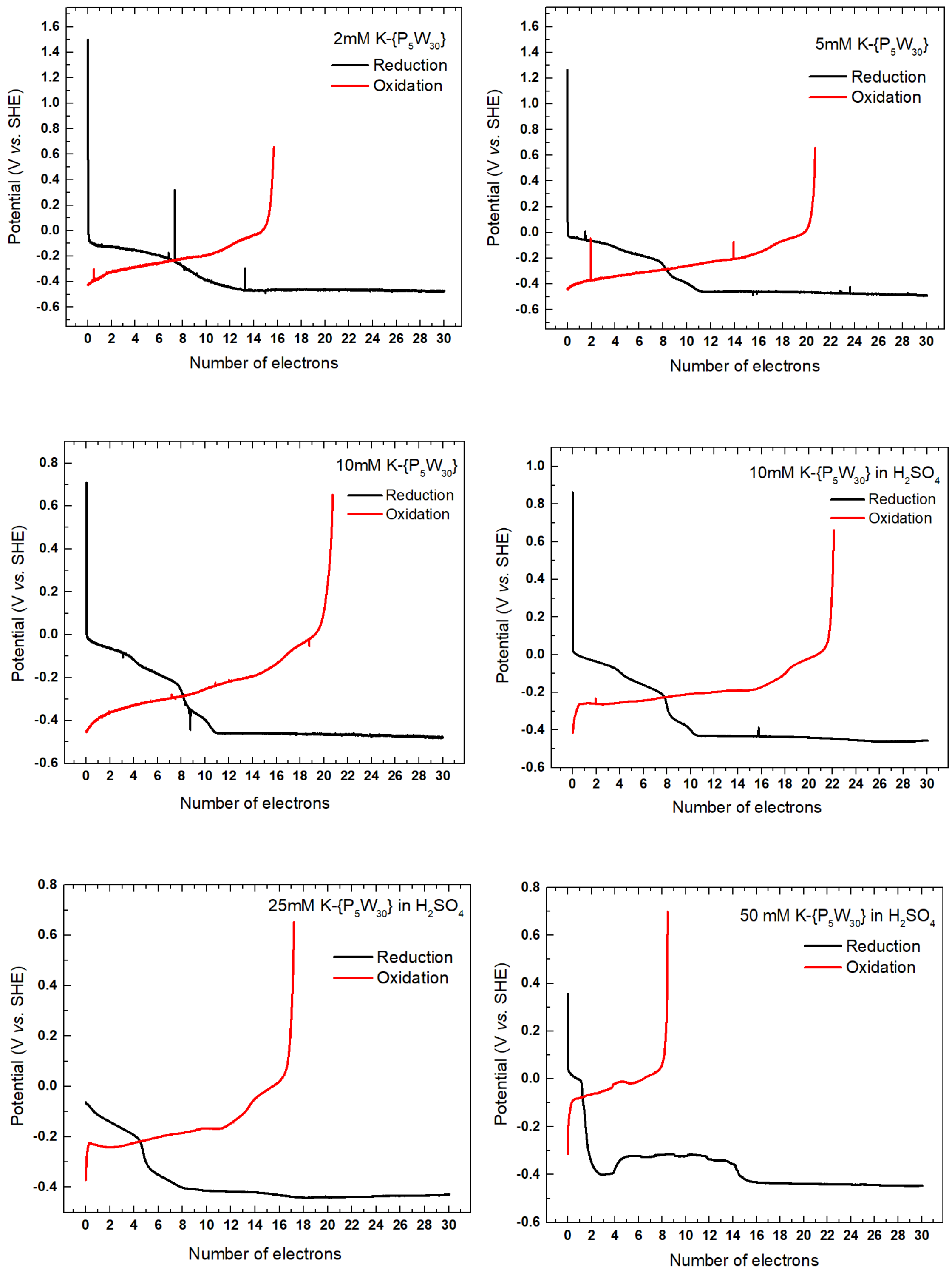

Figure S16: Reduction-oxidation curves at different concentrations (2 mM, 5 mM, 10 mM, 25 mM, 50 mM) corresponding to the data in Supplementary Table 4. All these experiments were performed under an atmosphere of Ar.

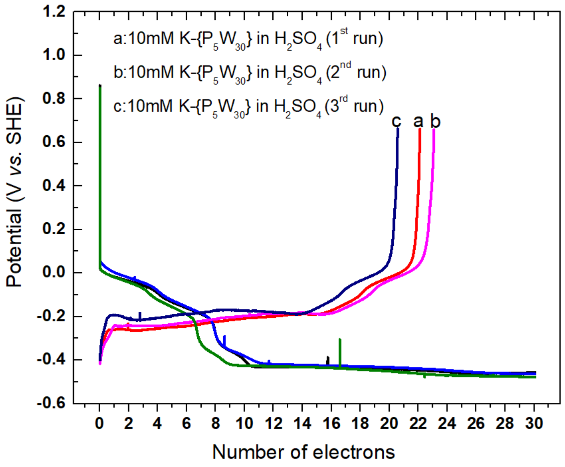

Figure S17: Reduction-oxidation curves under 10 mM in 0.2 M H_2SO_4 (run three times with the same solution)

This was conducted in the same solution for three times, a slight increase in the extracted number of electrons was observed on the second run. This also shows the recyclability of this K-{P_5W_{30}}.

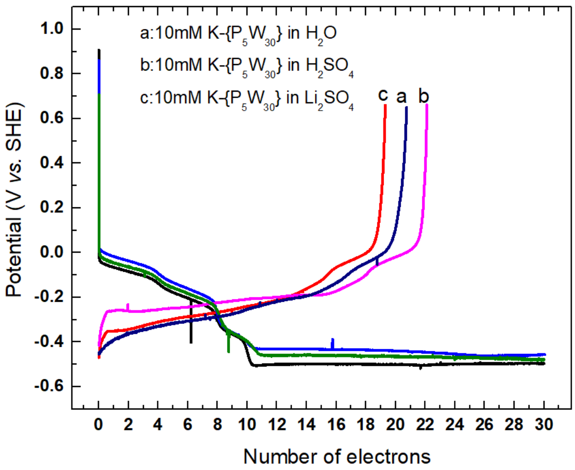

Figure S18: Reduction-oxidation curves under 10 mM in H_2O (20.7 e-), 0.2 M H_2SO_4 (22.1 e-) and 0.2 M Li_2SO_4 (19.3 e-)

This is a comparison of 10 mM K-{P_5W_{30}} in H_2O, 0.2 M H_2SO_4 and 0.2 M Li_2SO_4, showing the acid is a better supporting electrolyte.

4.3.2 Li-{P_5W_{30}}

Table S7: Comparison of the number of electrons that can be stored in Li-{P_5W_{30}} at different concentrations in H_2O/H_2SO_4. In all cases, the number of electrons used to initially reduce the Li-{P_5W_{30}} was set at 30 per cluster.

Conc. Li-{P_5W_{30}} (mM)	2	5	5	10	10	25	25
Volume (mL)	25	25	25	25	25	25	25
Supporting electrolyte	/	/	0.2 M H_2SO_4	0.2 M H_2SO_4	1 M H_2SO_4	1 M H_2SO_4	2 M H_2SO_4
Current density (mA cm^{-2})	2	5	5	10	10	25	25
Coulombic efficiency (%)	30.3	31.0	49.7	58.7	73.3	68.3	68.3
Number of electrons stored per cluster	9.1	9.3	14.9	17.6	22.0	20.5	20.5

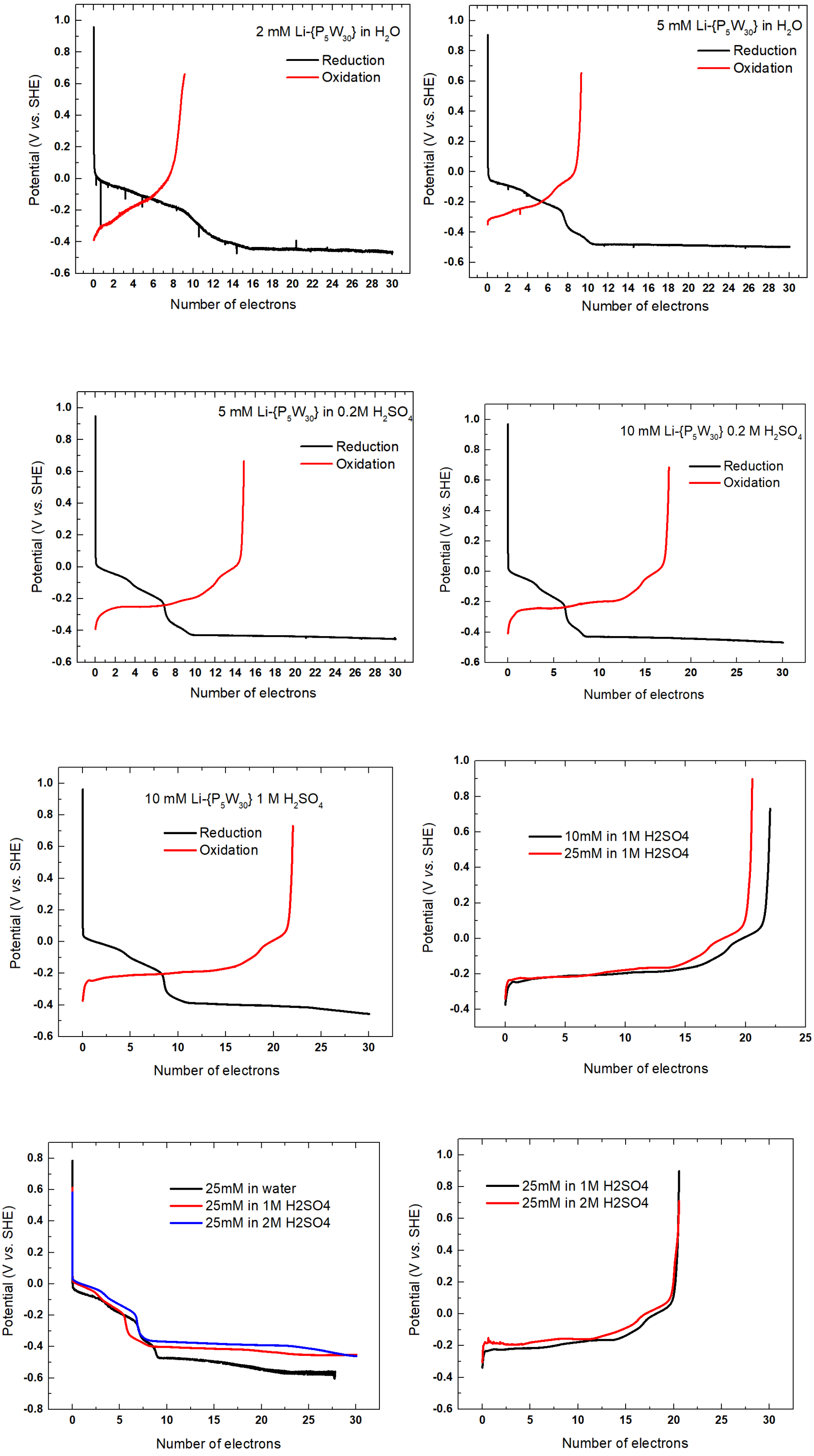

Figure S19: Reduction-oxidation curves at different concentrations (2 mM, 5 mM, 10 mM, 25 mM) corresponding to the data in Table S6. All these experiments were performed under an atmosphere of Ar.

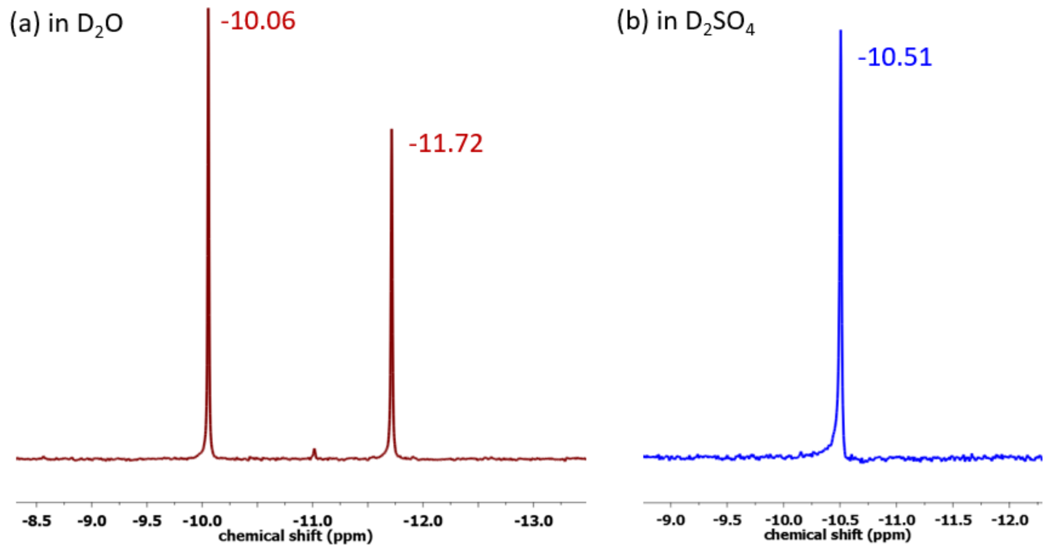

Figure S20: ^{31}P NMR of Li-{P_5W_{30}} in D_2O and D_2SO_4, showing Li-{P_5W_{30}} decompose in D_2O, while can maintain stability in D_2SO_4.

4.3.3 KLi-{P_8W_{48}}

Table S8: Comparison of the number of electrons that can be stored in KLi-{P_8W_{48}} at different concentrations in 1 M H_2SO_4. In all cases, the number of electrons used to initially reduce the KLi-{P_8W_{48}}was set at 30/32/48 per cluster.

Conc. KLi-{P_8W_{48}} (mM)	2	5	10	25	25	25	35
Volume (mL)	25	25	25	25	25	25	25
Current density (mA cm^{-2})	2	5	10	25	25	25	50
Coulombic efficiency (%)	44.7	57.3	80.0	89.7	84.5	/	82.3
Electrons input	30	30	30	30	32	48	32
Temperature	RT	RT	RT	70	70	60	70
Number of electrons stored per cluster	13.4	17.2	24.0	26.9	27.1	/	24.7

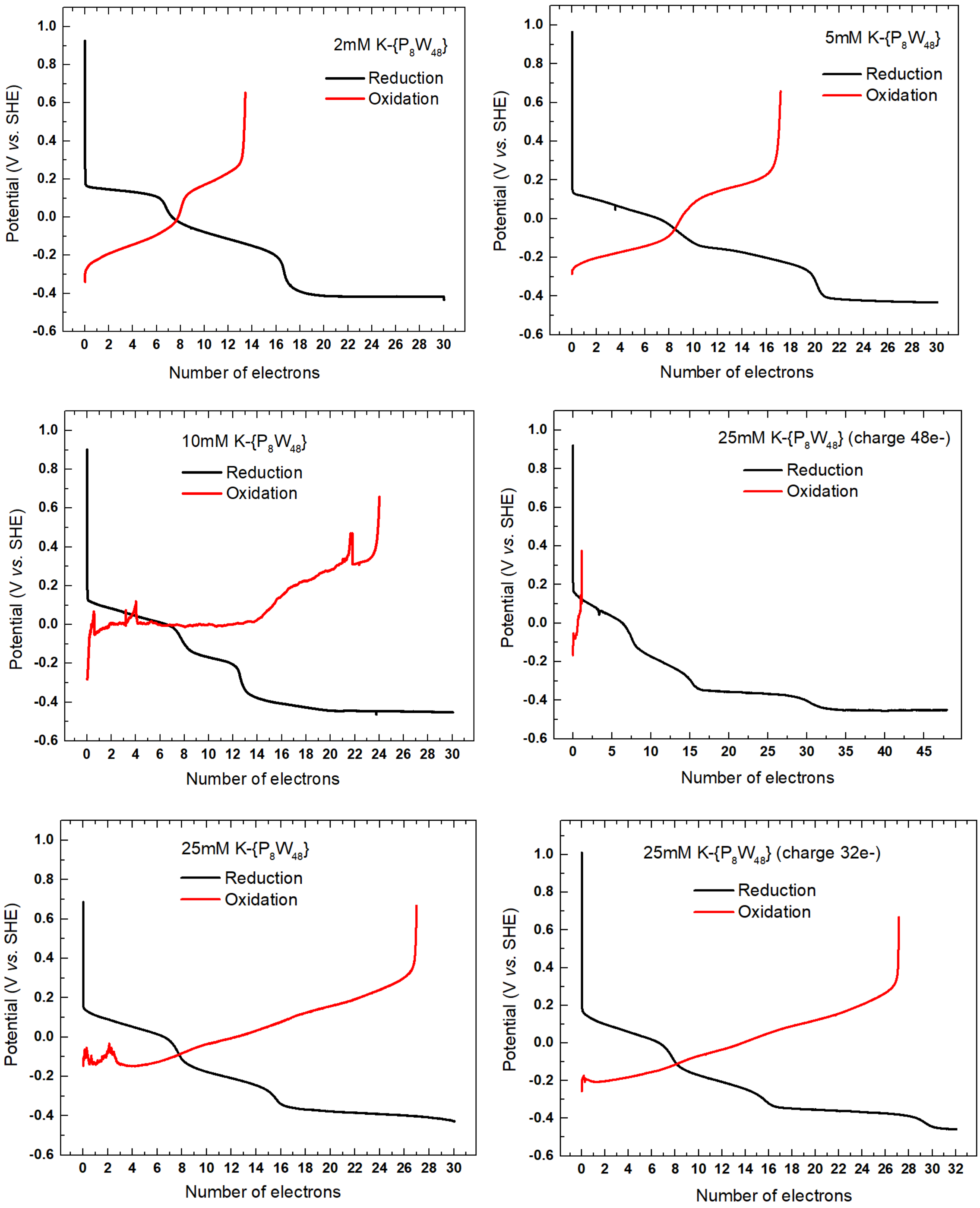

2mM K-{P8W48}
Reduction
Oxidation
Potential (V vs. SHE)
Number of electrons
5mM K-{P8W48}
Reduction
Oxidation
Potential (V vs. SHE)
Number of electrons
10mM K-{P8W48}
Reduction
Oxidation
Potential (V vs. SHE)
Number of electrons
25mM K-{P8W48} (charge 48e-)
Reduction
Oxidation
Potential (V vs. SHE)
Number of electrons
25mM K-{P8W48}
Reduction
Oxidation
Potential (V vs. SHE)
Number of electrons
25mM K-{P8W48} (charge 32e-)
Reduction
Oxidation
Potential (V vs. SHE)
Number of electrons

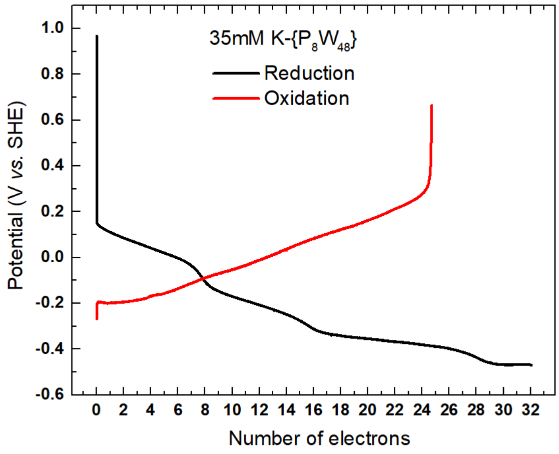

Figure S21: Reduction-oxidation curves at different concentrations (2 mM, 5 mM, 10 mM, 25 mM, 35 mM) corresponding to the data in Table S7. All these experiments were performed under an atmosphere of Ar. Unless noted, all solutions were charged as 30 e- per cluster.

4.3.4 LiNH4-{P8W48}

Table S9: Comparison of the number of electrons that can be stored in LiNH4-{P8W48} at different concentrations in 1 M H_2SO_4. In all cases, the number of electrons used to initially reduce the LiNH4-{P8W48} was set at 30 per cluster.

Conc. LiNH4-{P8W48} (mM)	10	25	35
Volume (mL)	25	25	25
Current density (mA cm^{-2})	10	25	50
Coulombic efficiency (%)	89.7	93.0	87.3
Electrons input	30	30	30
Number of electrons stored per cluster	26.9	27.9	26.2

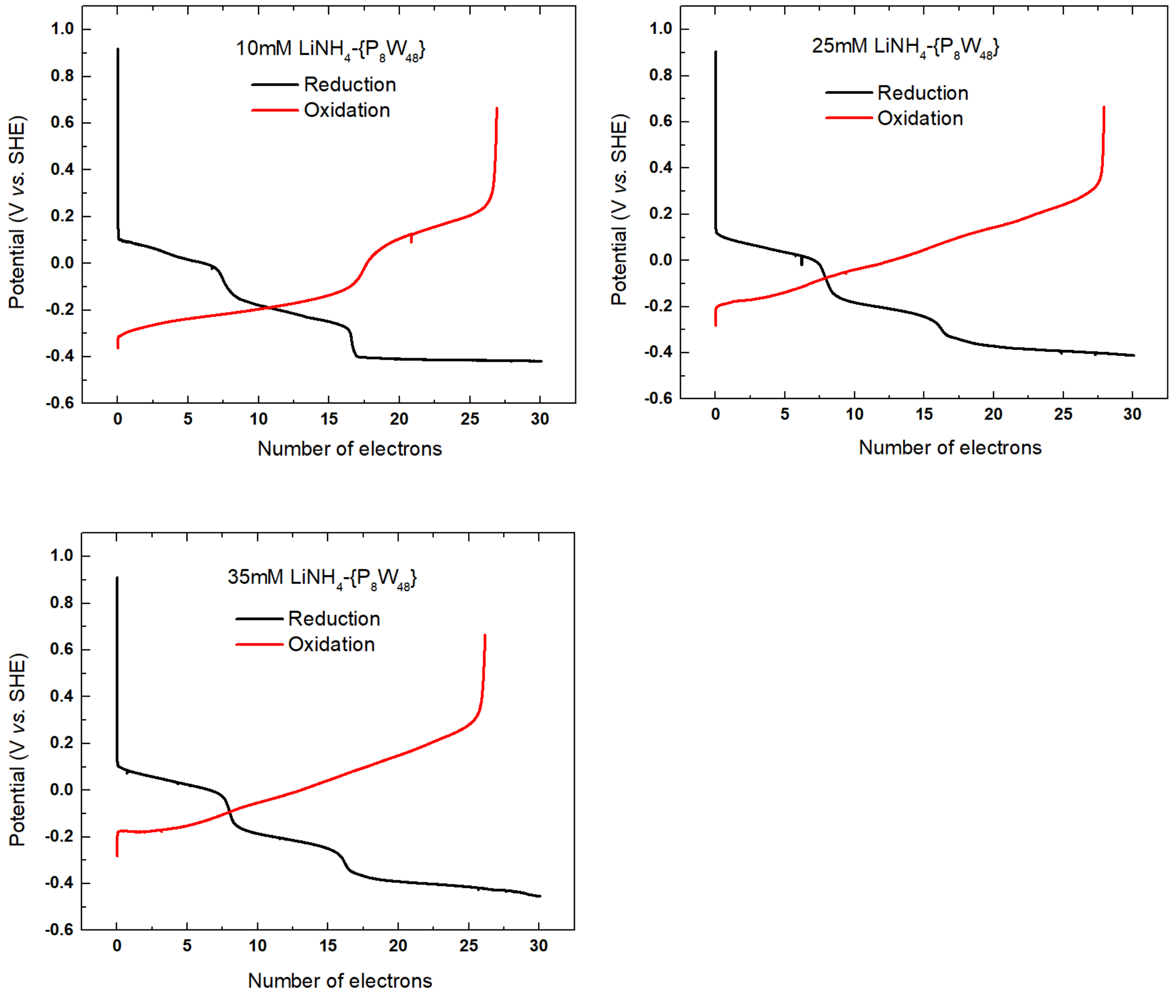

Figure S22: Reduction-oxidation curves at different concentrations (10 mM, 25 mM, 35 mM) corresponding to the data in Table S8. All these experiments were performed under an atmosphere of Ar.

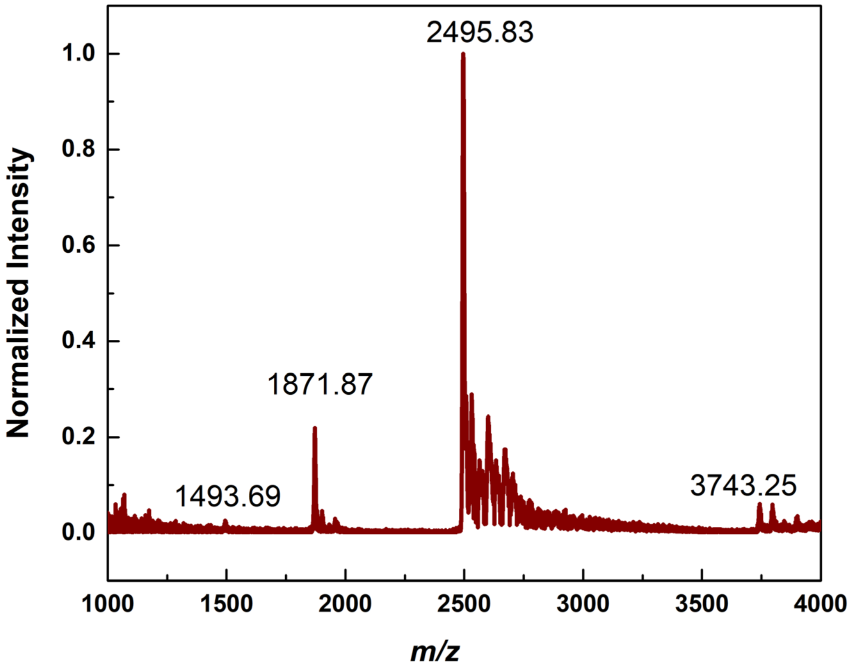

Figure S23: ESI-MS of $Li_{14}[NaP_5W_{30}O_{110}]\cdot 38H_2O$ in 1 M H_2SO_4 (diluted by methanol before measurement).

Table S10: Mass Spectrum peak table for $Li_{14}[NaP_5W_{30}O_{110}]\cdot 38H_2O$ in 1 M H_2SO_4 shown in Figure S23

Z	m/z (Obs)	m/z (Cal)	Assignment
5-	1493.69	1497.16	$[Li_4H_5NaP_5W_{30}O_{110}]^{5-}$
4-	1871.87	1871.70	$[Li_4H_6NaP_5W_{30}O_{110}]^{4-}$
3-	2495.83	2495.94	$[Li_4H_7NaP_5W_{30}O_{110}]^{3-}$
	2509.13	2507.85	$[Li_4H_7NaP_5W_{30}O_{110}]^{3-}\cdot(H_2O)_2$
	2530.46	2531.96	$[Li_4H_7NaP_5W_{30}O_{110}]^{3-}\cdot(H_2O)_6$
	2564.14	2563.97	$[Li_5H_6NaP_5W_{30}O_{110}]^{3-}\cdot(H_2O)_{11}$
	2602.45	2601.98	$[Li_6H_5NaP_5W_{30}O_{110}]^{3-}\cdot(H_2O)_{17}$
	2634.45	2633.98	$[Li_7H_4NaP_5W_{30}O_{110}]^{3-}\cdot(H_2O)_{22}$
	2643.77	2641.96	$[Li_8H_3NaP_5W_{30}O_{110}]^{3-}\cdot(H_2O)_{23}$
	2669.76	2070.01	$[Li_7H_4NaP_5W_{30}O_{110}]^{3-}\cdot(H_2O)_{28}$
	2702.77	2702.02	$[Li_8H_3NaP_5W_{30}O_{110}]^{3-}\cdot(H_2O)_{33}$
	2748.42	2748.00	$[Li_{10}HNaP_5W_{30}O_{110}]^{3-}\cdot(H_2O)_{40}$
	2782.41	2782.06	$[Li_9H_2NaP_5W_{30}O_{110}]^{3-}\cdot(H_2O)_{46}$

2-	3743.28	3744.41	$[Li_4H_8NaP_5W_{30}O_{110}]^{2-}$
	3796.72	3798.46	$[Li_4H_8NaP_5W_{30}O_{110}]^{2-}\cdot(H_2O)_6$

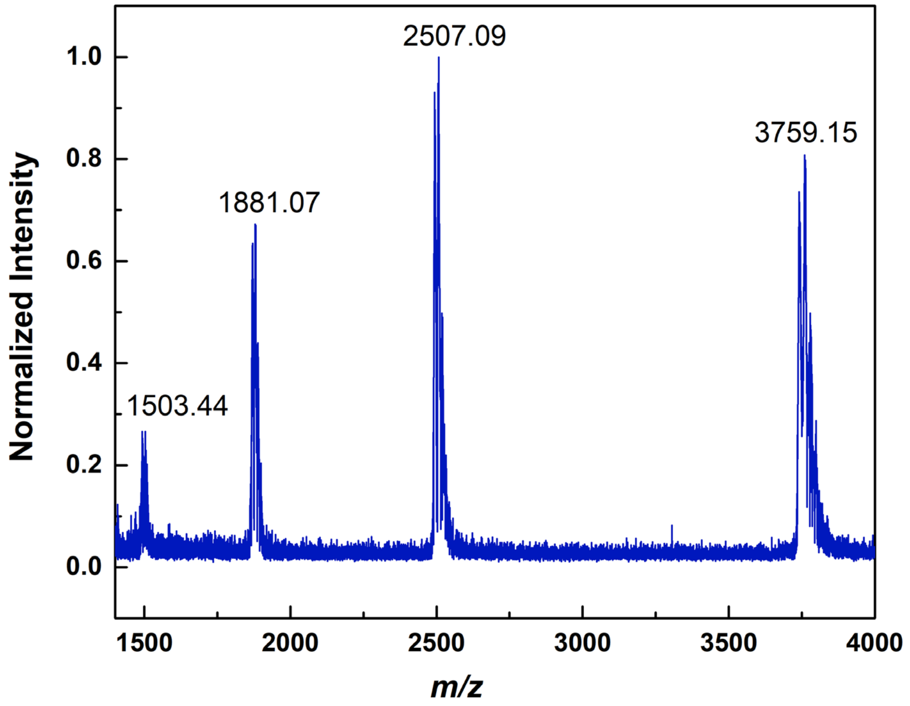

Figure S24: ESI-MS of $K_{14}[NaP_5W_{30}O_{110}]\cdot 22H_2O$ in 1 M H_2SO_4 (diluted by water before measurement).

Table S11: Mass Spectrum peak table for $K_{14}[NaP_5W_{30}O_{110}]$ in acid shown in Figure S24

z	m/z (Obs)	m/z (Cal)	Assignment
5-	1495.06	1496.81	$[H_8Na_2P_5W_{30}O_{110}]^{5-}$
	1503.44		
4-	1870.81	1871.26	$[H_9Na_2P_5W_{30}O_{110}]^{4-}$
	1881.07	1880.79	$[KH_8Na_2P_5W_{30}O_{110}]^{4-}$
	1890.29	1890.31	$[K_2H_7Na_2P_5W_{30}O_{110}]^{4-}$
3-	2493.44	2495.36	$[H_{10}Na_2P_5W_{30}O_{110}]^{3-}$
	2507.09	2508.05	$[KH_9Na_2P_5W_{30}O_{110}]^{3-}$
	2520.42	2520.75	$[K_2H_8Na_2P_5W_{30}O_{110}]^{3-}$
2-	3742.18	3743.54	$[H_{11}Na_2P_5W_{30}O_{110}]^{2-}$
	3759.15	3762.58	$[KH_{10}Na_2P_5W_{30}O_{110}]^{2-}$

3778.66	3781.63	$[K_2H_9Na_2P_5W_{30}O_{110}]^{2-}$

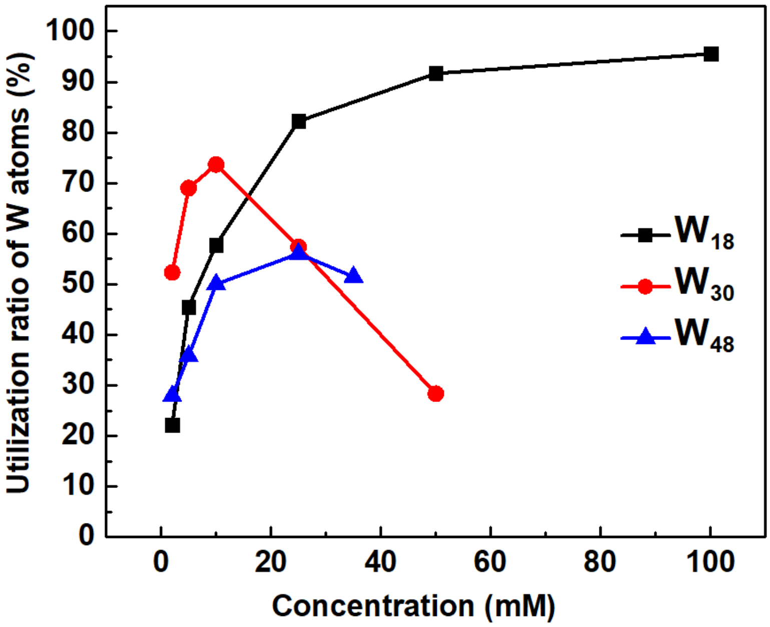

Figure S25: A comparison of electrochemical performance between Li-$\{P_2W_{18}\}$, K-$\{P_5W_{30}\}$ and KLi-$\{P_8W_{48}\}$ regarding utilization ratio of W atoms.

$\{P_8W_{48}\}$ has the lowest utilization rate of W, only part of W in the structure are redox active and participate in the reduction/oxidation process.

5. NMR and ESI-MS analysis of reduction/reoxidation

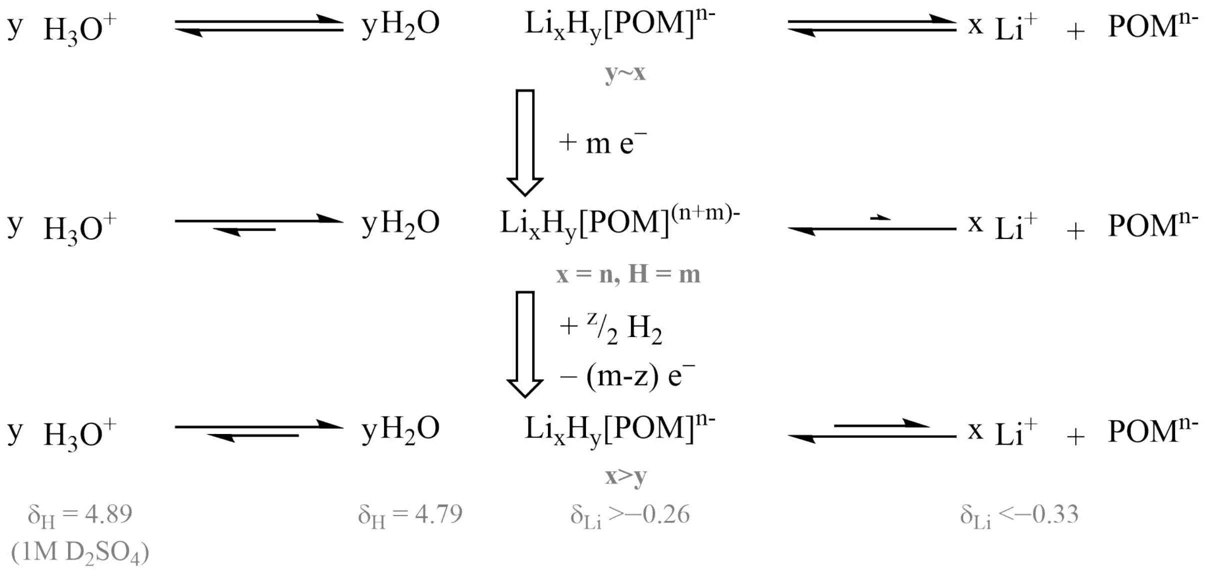

Figure S26: Equilibrium position of H_3O^+ and Li^+ during reduction and reoxidation and how this affects NMR shift observed.

The performance limit of Li-{P_5W_{30}} was analyzed by NMR, MS and GC.

Table S12: a series of experiment of Li-{P_5W_{30}} to study the performance limit

	Charge /cluster	GC	Original		Reduced		Reoxidized		From MS/NMR
			NMR	MS	NMR	MS	NMR	MS	
Li-{P_5W_{30}} 10mM in 1 M H_2SO_4	30 e^-	√		√		√		√	No decomposition observed
Li-{P_5W_{30}} 10mM in 1 M D_2SO_4	30 e-		√	√	√	√	√	√	Decomposition observed
Li-{P_5W_{30}} 10mM in 1M H_2SO_4	24 e-	√	√	√	√	√	√	√	No decomposition observed
Li-{P_5W_{30}} 10mM in 1 M H_2SO_4	20 e-	√	√	√	√	√	√	√	No decomposition observed

Note: √ means this technique was applied

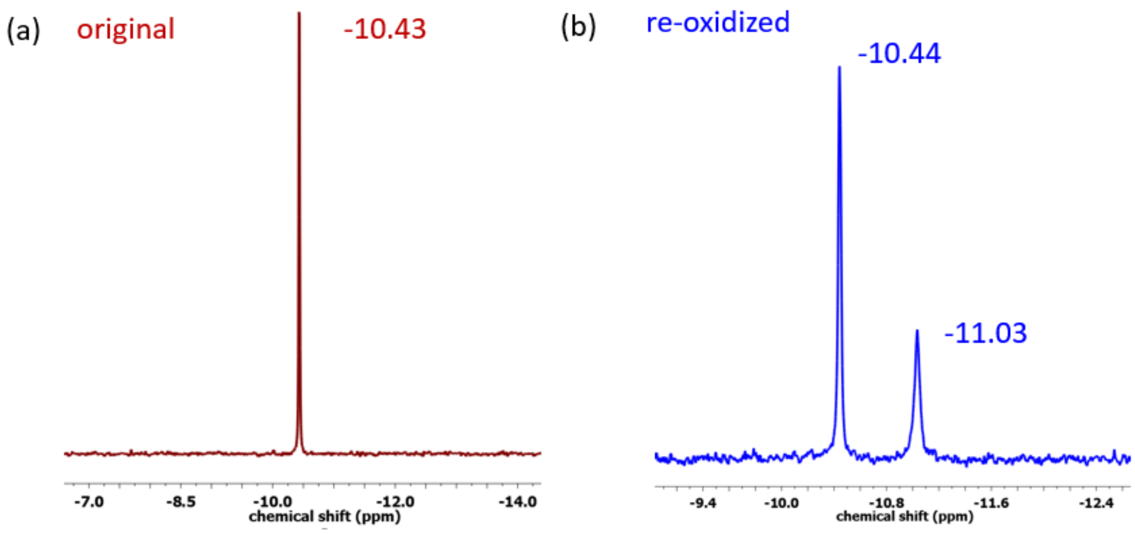

Figure S27: ^{31}P NMR of 10 mM of Li-{P_5W_{30}} in 1 M D_2SO_4 charge 30 e- per polyoxometalate cluster (a) original (b) re-oxidized, showing a decomposition using D_2SO_4 as supporting electrolyte for electrochemical measurement.

Effect of deuterium (D_2SO_4) in electrochemical tests

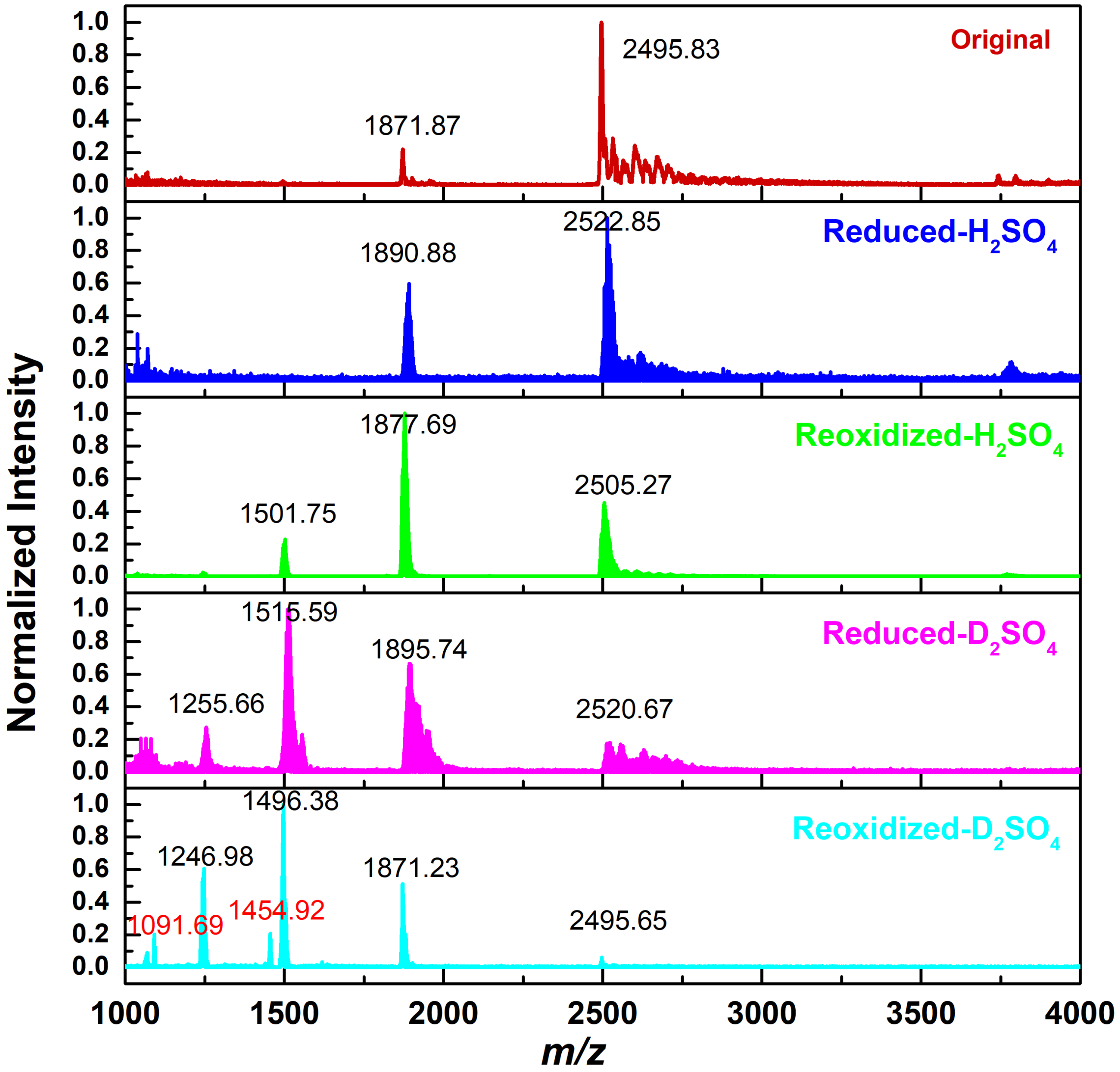

Figure S28: Mass spectrum of samples of Li-{P_5W_{30}} solution (a) original, taken from a 10 mM of Li-{P_5W_{30}} solution that was (b) reduced by 30 e- per polyoxometalate cluster in H_2SO_4 (c) re-oxidized in H_2SO_4 was (d) reduced by 30 e- per polyoxometalate cluster in D_2SO_4 (e) re-oxidized in D_2SO_4 and then injected into the mass spectrometer.

The reduced sample was taken out immediately after charging finish and protected with Ar to avoid oxidation by air, before injection it was also diluted with degassed methanol. The original and re-oxidized one was also after dilution with degassed methanol.

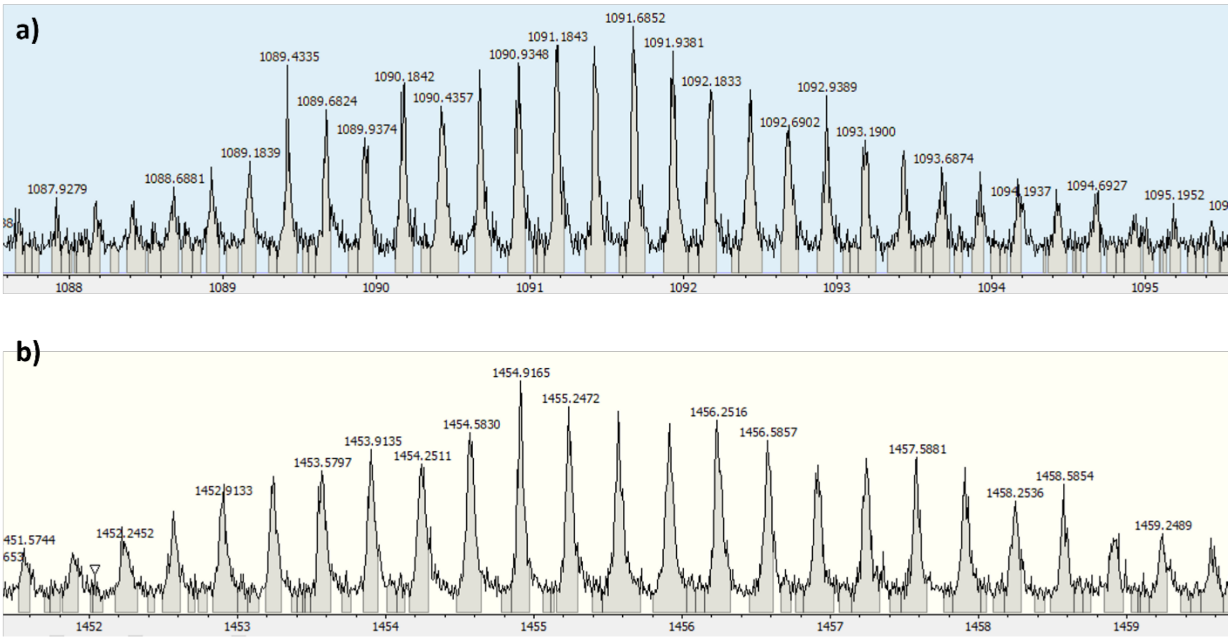

Figure S29: Peaks around 1091.69 and 1454.92 *m/z* in mass spectrum of samples of Li-$\{P_5W_{30}\}$ in re-oxidized D_2SO_4 in Figure S28, can be assigned as $[H_2P_2W_{18}O_{62}]^{4-}$ and $[H_3P_2W_{18}O_{62}]^{3-}$ respectively.

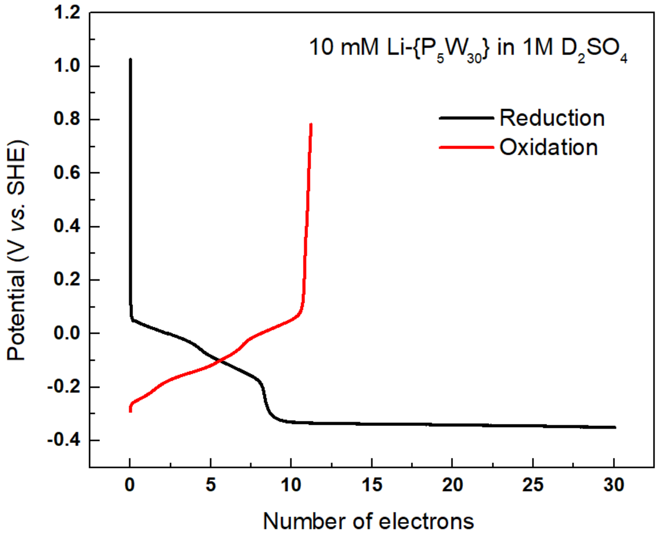

Figure S30: Reduction-oxidation curves under 10 mM Li-$\{P_5W_{30}\}$ in 1 M D_2SO_4, charge 30 e- per polyoxometalate cluster

6. Concentration effect of Li-{P_2W_{18}} and Li-{P_5W_{30}} and LiNH$_4$-{P_8W_{48}}

The various concentration of the non-reduced polyoxotungstate solution was investigated by ^{1}H, ^{7}Li and ^{31}P NMR study. All the reduced samples were taken out immediately after charging finish and protected with Ar to avoid oxidation by air. For D_2SO_4 ones, samples were directly taken and sealed with Ar for NMR tests. For H_2SO_4 ones, a few drops of D_2O ($V_{sample\ solution}$: V_{D_2O}= 500 uL : 10 uL) were added for locking in NMR measurement, and then sealed with Ar.

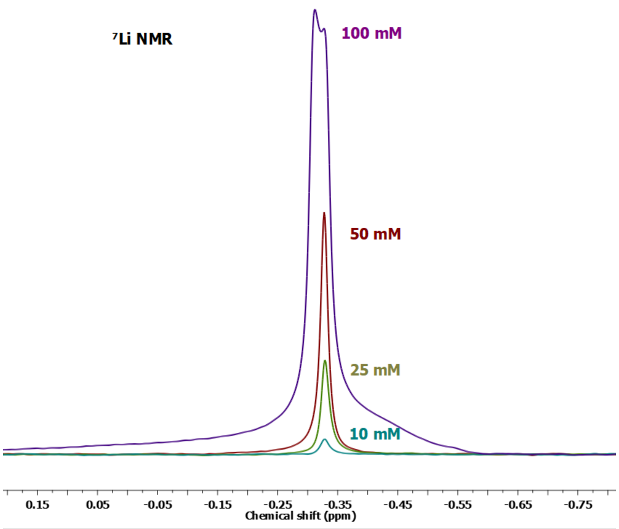

Figure S31: ^{7}Li NMR of LiCl (10 mM, 25 mM, 50 mM and 100 mM of solution) in 1 M D_2SO_4

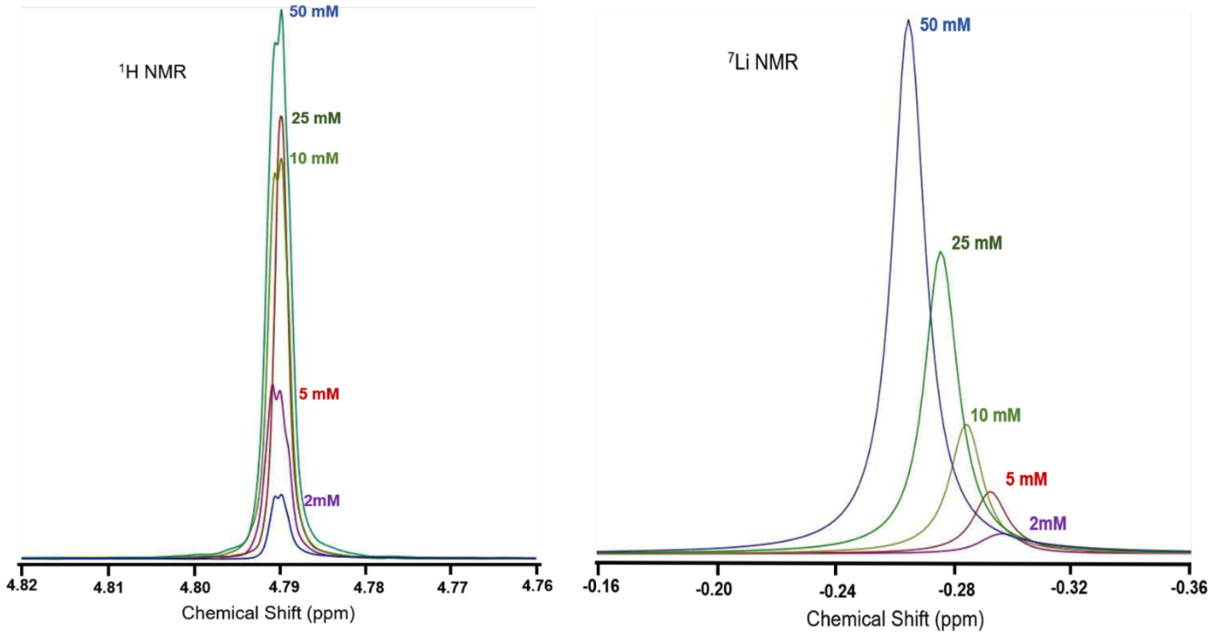

Figure S32: [1]H and [7]Li of non-reduced Li-P_2W_{18} (2 mM, 5 mM, 10 mM, 25 mM and 50 mM of solution) in 1 M D_2SO_4

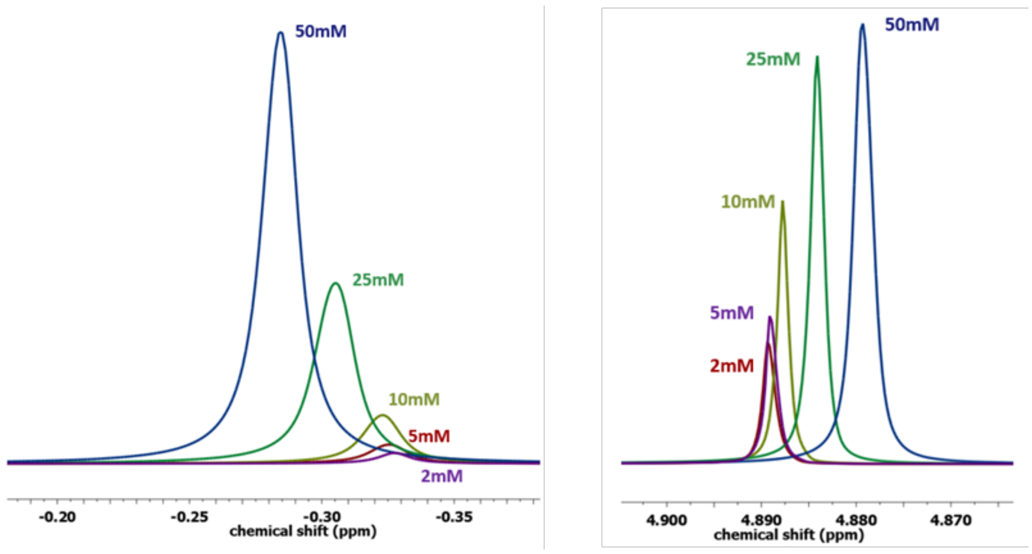

Figure S33: ^{7}Li and ^{1}H NMR of original solution of 2 mM, 5 mM, 10 mM, 25 mM and 50 mM of Li-{P_5W_{30}} in 1 M D_2SO_4

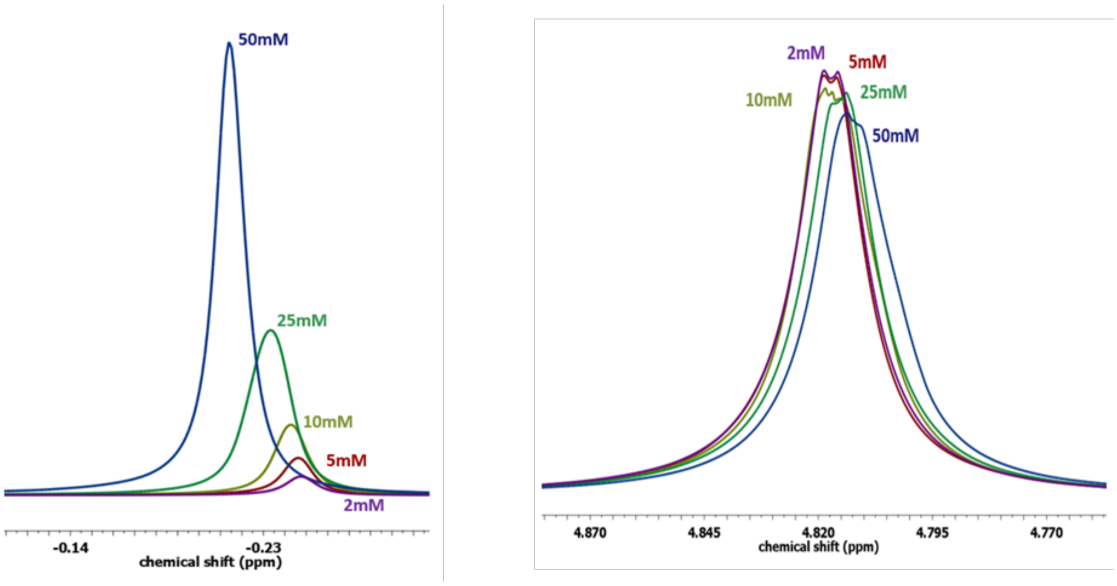

Figure S34: ^{7}Li and ^{1}H NMR of original solution of 2 mM, 5 mM, 10 mM, 25 mM and 50 mM of Li-$\{P_5W_{30}\}$ in 1 M H_2SO_4

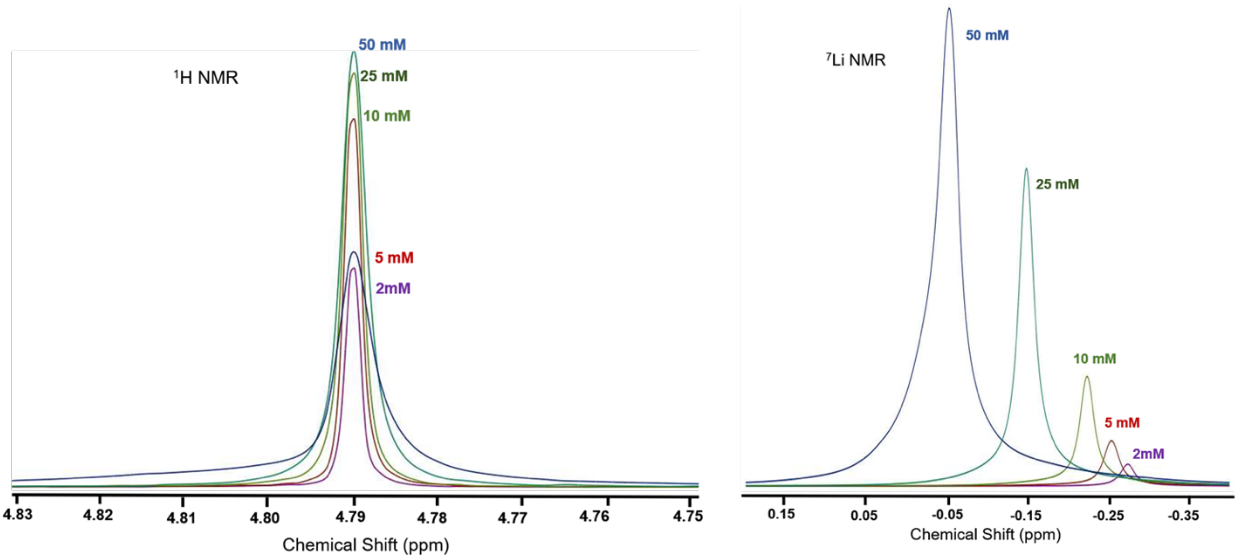

Figure S35: ^{1}H and ^{7}Li of non-reduced $LiNH_4$-$\{P_8W_{48}\}$ (2 mM, 5 mM, 10 mM, 25 mM and 50 mM of solution) in 1 M D_2SO_4

7. Performance limit analysis

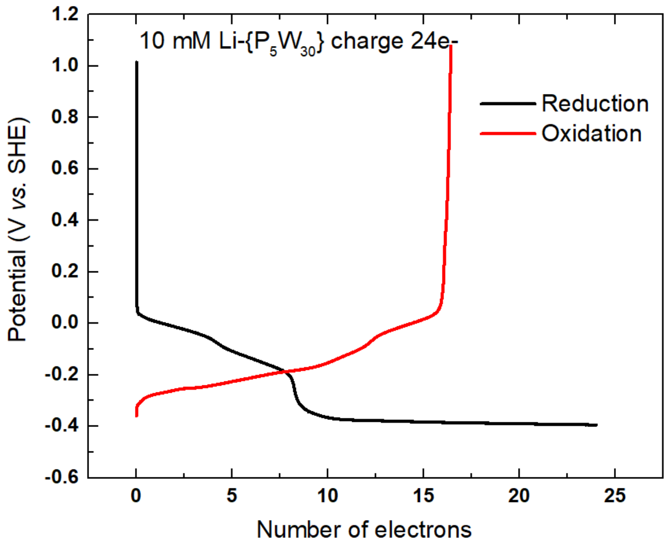

Figure S36: Reduction-oxidation curves under 10 mM Li-{P_5W_{30}} in 1 M H_2SO_4, charge 24 e- per polyoxometalate cluster

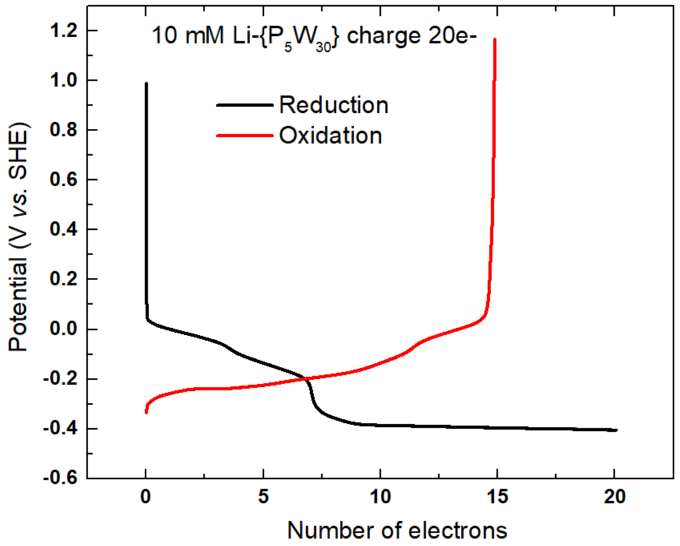

Figure S37: Reduction-oxidation curves under 10 mM Li-{P_5W_{30}} in 1 M H_2SO_4, charge 20 e- per polyoxometalate cluster

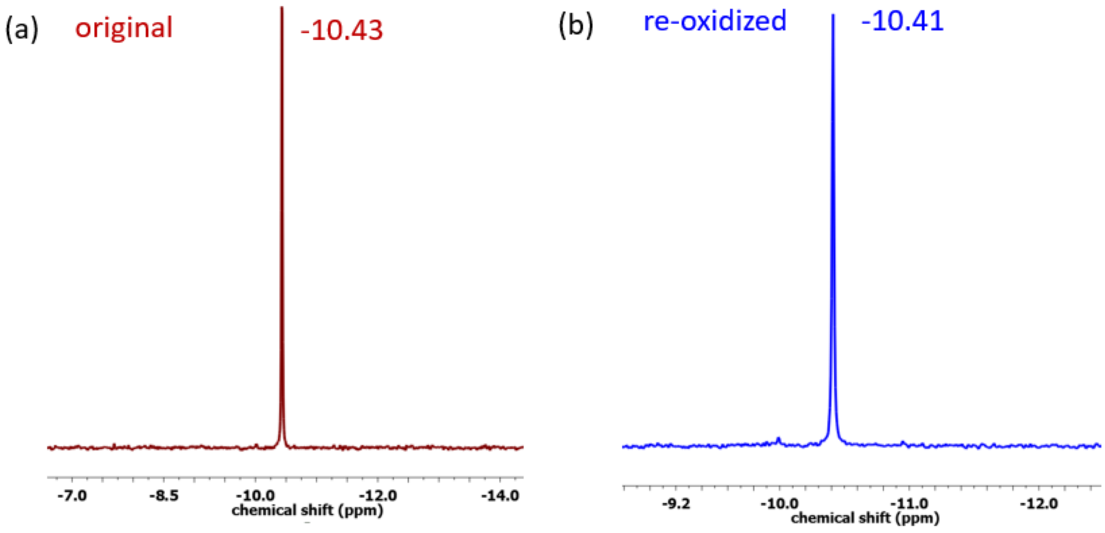

Figure S38: ^{31}P NMR of 10 mM Li-{P_5W_{30}} in 1 M H_2SO_4 charge 20 e- per polyoxometalate cluster (a) original (b) re-oxidized, showing no decomposition after reduction.

ESI-MS analysis

The reduced sample was taken out immediately after charging finish and protected with Ar to avoid oxidation by air, before injection it was also diluted with degassed methanol. The original and re-oxidized one was also after dilution with degassed methanol. The peaks for original one has been analysed in supplementary Table 3. Through assignment of the peaks (Table S12 and S13), we found after reduction {P_5W_{30}} was protonated and after re-oxidization, there are more Li^+ coordinated in the structure than in the original status (Table S9).

Table S13: Mass Spectrum peak table for Li-{P_5W_{30}} (reduced) shown in Figure 6

z	m/z (Obs)	m/z (Cal)	Assignment
4-	1890.88	1891.13	$[H_{28}Li_{12}NaP_5W_{30}O_{110}]^{4-}$
3-	2522.85	2522.17	$[H_{29}Li_{12}NaP_5W_{30}O_{110}]^{3-}$

Table S14: Mass Spectrum peak table for Li-{P_5W_{30}} (reoxidized) shown in Figure 6

Z	m/z (Obs)	m/z (Cal)	Assignment

6-	1244.28	1244.50	$[LiH_7NaP_5W_{30}O_{110}]^{6-}$
5-	1501.75	1501.91	$[Li_8HNaP_5W_{30}O_{110}]^{5-}$
4-	1877.69	1877.64	$[Li_8H_2NaP_5W_{30}O_{110}]^{4-}$
3-	2505.27	2505.83	$[Li_9H_2NaP_5W_{30}O_{110}]^{3-}$

Table S15: Mass Spectrum peak table for the spectrum shown in Figure S28 (reduced in D_2SO_4)

Z	m/z (Obs)	m/z (Cal)	Assignment
6-	1255.66	1258.17	$[Li_4D_{34}NaP_5W_{30}O_{110}]^{6-}$
5-	1515.59	1515.14	$[Li_9D_{30}NaP_5W_{30}O_{110}]^{5-}$
4-	1895.74	1895.66	$[Li_{10}D_{30}NaP_5W_{30}O_{110}]^{4-}$
3-	2520.67	2521.64	$[Li_6D_{35}NaP_5W_{30}O_{110}]^{3-}$

Table S16: Mass Spectrum peak table for the spectrum shown in Figure S28 (reoxidized in D_2SO_4)

Z	m/z (Obs)	m/z (Cal)	Assignment
6-	1246.98	1246.48	$[Li_2D_6NaP_5W_{30}O_{110}]^{6-}$
5-	1496.38	1497.17	$[Li_3D_6NaP_5W_{30}O_{110}]^{5-}$
4-	1871.23	1871.97	$[Li_3D_7NaP_5W_{30}O_{110}]^{4-}$
3-	2495.65	2496.62	$[Li_3D_8NaP_5W_{30}O_{110}]^{3-}$

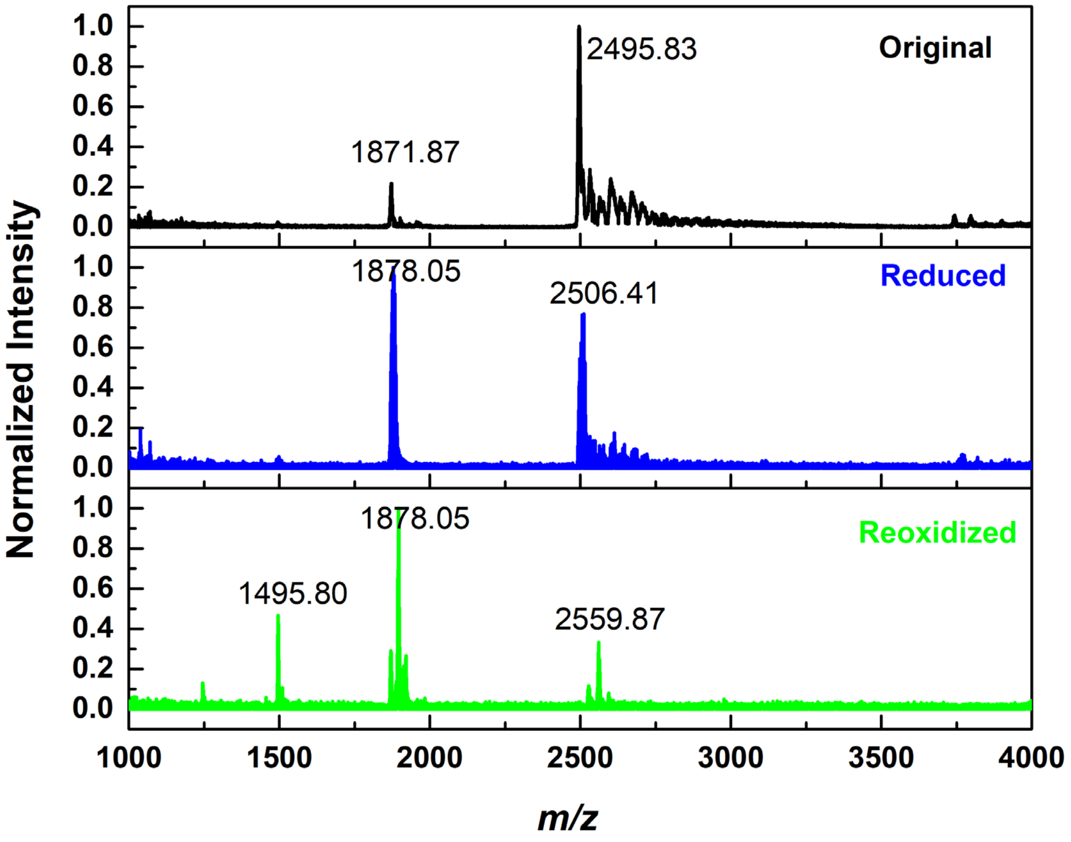

Figure S39: Mass spectrum of samples of the polyoxometalate solution: (a) original, taken from a 10 mM solution of Li-{P_5W_{30}} that was (b) reduced by 24 e- (c)re-oxidized and then injected into the mass spectrometer. The reduced sample was taken out immediately after charging finish and protected with Ar to avoid oxidation by air, before injection it was also diluted with degassed methanol. The original and re-oxidized one was also after dilution with degassed methanol.

Table S17: Mass Spectrum peak table for the spectrum of shown in Figure S39 (reduced)

Z	m/z (Obs)	m/z (Cal)	Assignment
4-	1878.05	1877.64	$[Li_4H_{30}NaP_5W_{30}O_{110}]^{4-}$
3-	2506.41	2505.98	$[Li_5H_{30}NaP_5W_{30}O_{110}]^{3-}$

Table S18: Mass Spectrum peak table for the spectrum shown in Figure S39(reoxidized)

Z	m/z (Obs)	m/z (Cal)	Assignment
5-	1495.80	1495.97	$[Li_3H_6NaP_5W_{30}O_{110}]^{5-}$
4-	1878.05	1877.64	$[Li_8H_2NaP_5W_{30}O_{110}]^{4-}$
3-	2560.41	2559.87	$[Li_9H_2NaP_5W_{30}O_{110}]^{3-}\cdot(H_2O)_9$

Note: during the operation of this group experiment, there are some water backflow from a sequencing GC setup when taking samples from the electrochemical vial for the deoxidized one, leading to some possible shift on the *m/z* position, but it still show the stable structure of $[NaP_5W_{30}O_{110}]^{14-}$

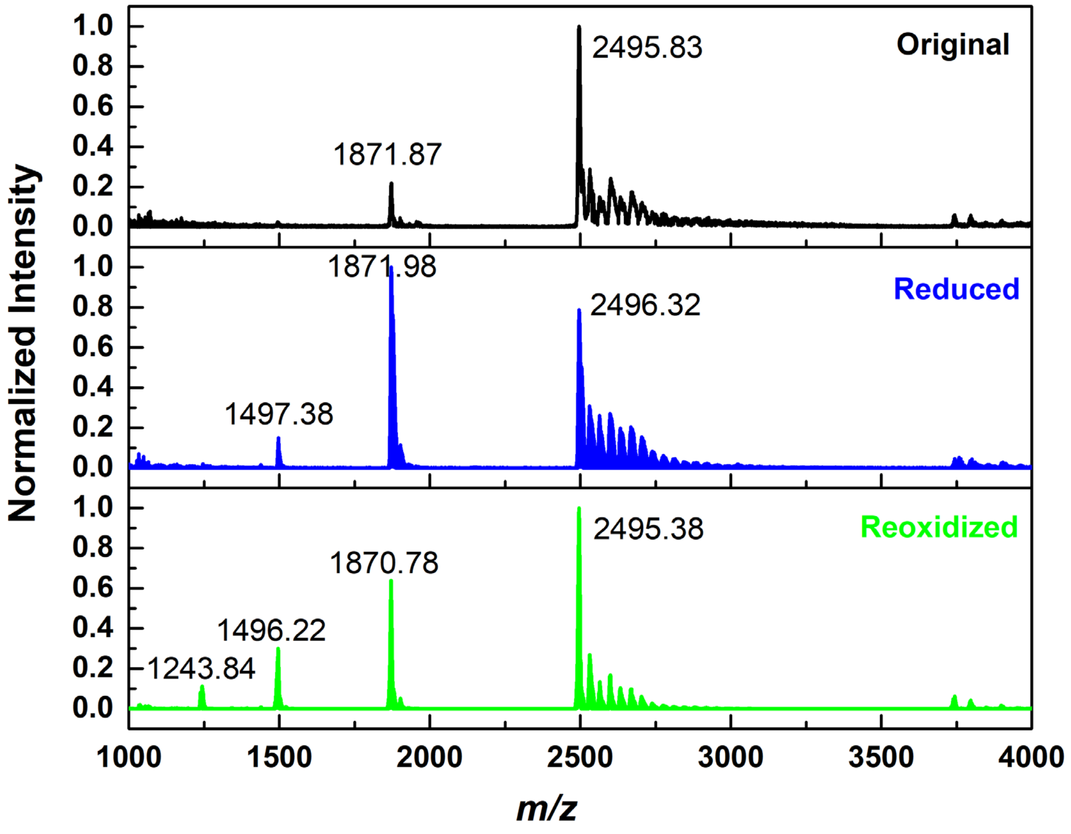

Figure S40: Mass spectrum of samples of the polyoxometalate solution (a) original, taken from a 10 mM solution of Li-$\{P_5W_{30}\}$ that was (b) reduced by 20 e- (c) re-oxidized and then injected into the mass spectrometer. The reduced sample was taken out immediately after charging finish and protected with Ar to avoid oxidation by air, before injection it was diluted

with degassed methanol. The original and re-oxidized one was also after dilution with degassed methanol.

Table S19: Mass Spectrum peak table for the spectrum of shown in Figure S40 (reduced)

Z	m/z (Obs)	m/z (Cal)	Assignment
5-	1497.38	1497.63	$[LiH_{28}NaP_5W_{30}O_{110}]^{5-}$
4-	1871.98	1872.29	$[LiH_{29}NaP_5W_{30}O_{110}]^{4-}$
3-	2496.32	2496.72	$[LiH_{30}NaP_5W_{30}O_{110}]^{3-}$

Table S20: Mass Spectrum peak table for the spectrum shown in Figure S40 (reoxidized)

Z	m/z (Obs)	m/z (Cal)	Assignment
6-	1243.84	1244.50	$[LiH_7NaP_5W_{30}O_{110}]^{6-}$
5-	1496.22	1495.97	$[Li_3H_6NaP_5W_{30}O_{110}]^{5-}$
4-	1870.78	1870.22	$[Li_3H_7NaP_5W_{30}O_{110}]^{4-}$
3-	2495.38	2495.94	$[Li_4H_7NaP_5W_{30}O_{110}]^{3-}$

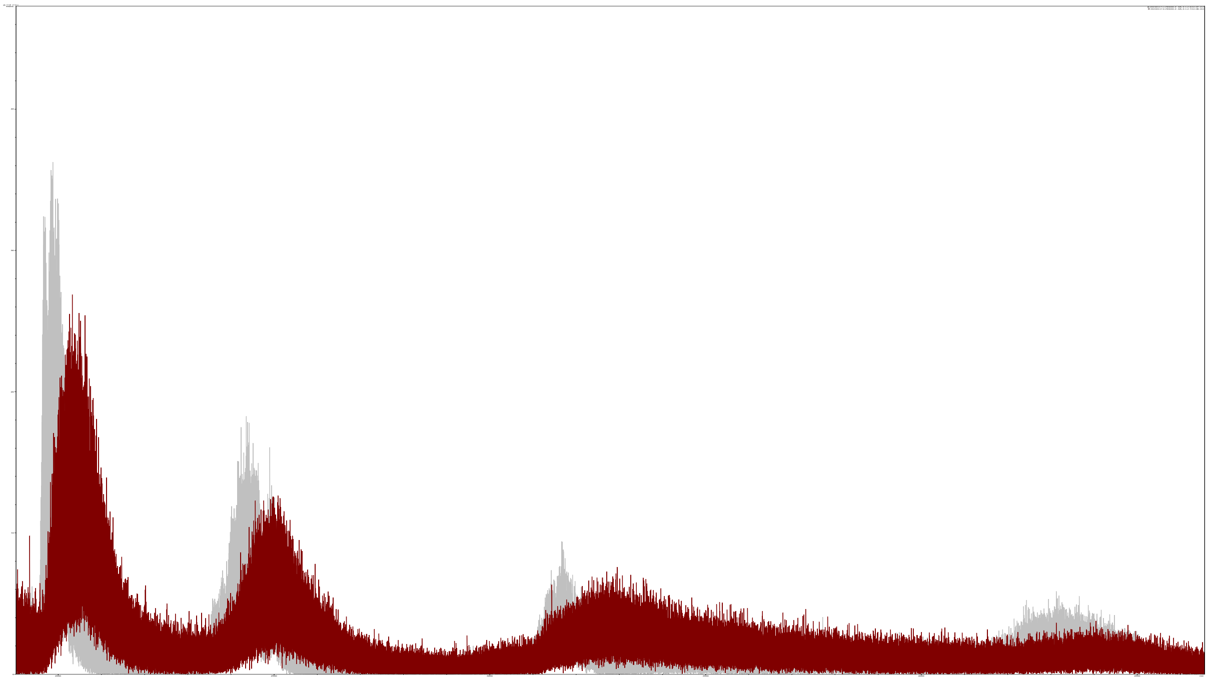

Figure S41: Overlaid ESI-MS of $Li(NH_4)$-P_8W_{48} and KLi-P_8W_{48} (dissolved in 1M H_2SO_4 and diluted with water) showing broad, non-overlapping peak envelopes due to a wide variation in cation incorporation in solution.

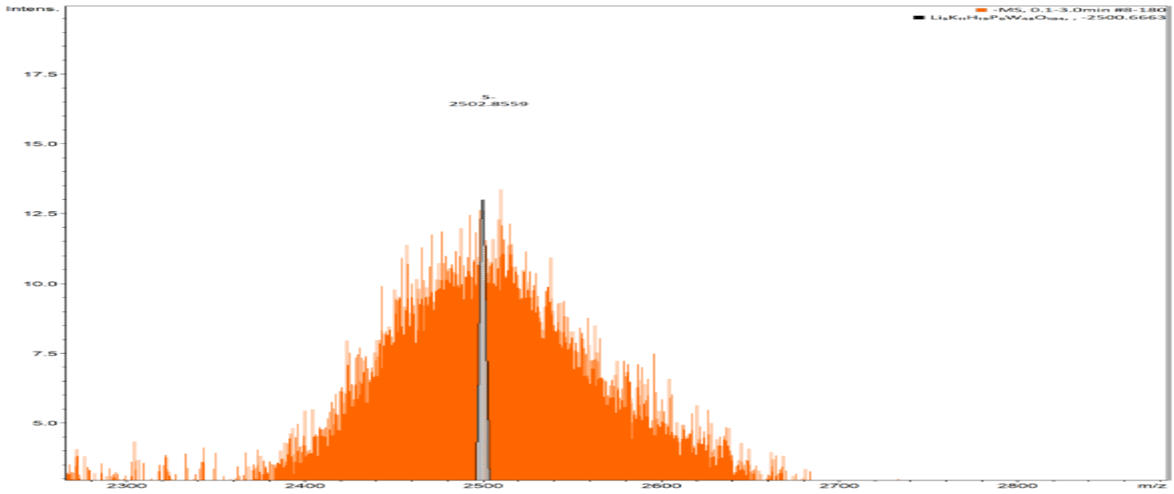

Figure S42: ESI-MS KLi-P_8W_{48} (dissolved in 1M H_2SO_4 and diluted with water) with exemplar simulated ion $K_{11}Li_5H_{19}$-$\{P_8W_{48}\}^{5-}$ (2500.6 m/z).

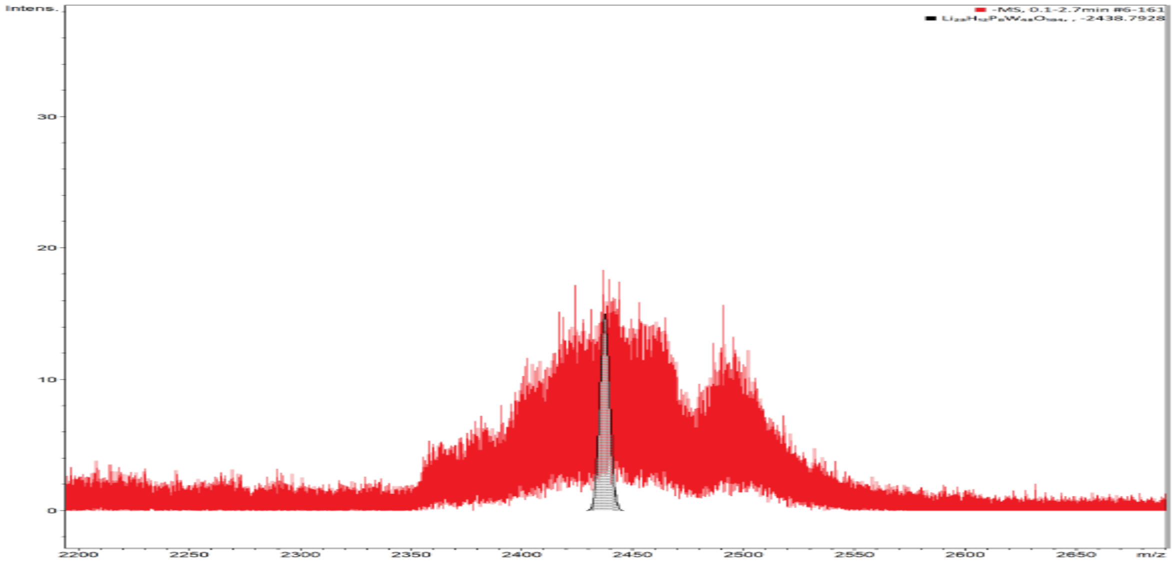

Figure S43: ESI-MS Li(NH_4)-P_8W_{48} (dissolved in 1M H_2SO_4 and diluted with water) with exemplar simulated ion $Li_{23}H_{12}$-$\{P_8W_{48}\}^{5-}$ (2438.79 m/z).

8. References

1. L. Farrugia, *J. Appl. Crystallogr.*, 1999, **32**, 837-838.
2. R. H. Blessing, *Acta Cryst.* , 1995, **AS1**, 33-38.
3. A. Hayashi, T. Haioka, K. Takahashi, B. S. Bassil, U. Kortz, T. Sano and M. Sadakane, *Z. Anorg. Allg. Chem.*, 2015, **641**, 2670-2676.
4. M. J. Turo, L. Chen, C. E. Moore and A. M. Schimpf, *J. Am. Chem. Soc.*, 2019, **141**, 4553-4557.
5. R. Contant and A. Teze, *Inorg. Chem.*, 1985, **24**.
6. J. J. Chen, M. D. Symes and L. Cronin, *Nature Chem.*, 2018, **10**, 1042-1047.
7. M. Wang, F. Zhao and S. Dong, *J. Phys. Chem. B*, 2004, **108**, 5.
